# OSMAC-Based Discovery and Biosynthetic Gene Clusters Analysis of Secondary Metabolites from Marine-Derived *Streptomyces globisporus* SCSIO LCY30

**DOI:** 10.3390/md22010021

**Published:** 2023-12-28

**Authors:** Yanqing Li, Naying Gong, Le Zhou, Zhijie Yang, Hua Zhang, Yucheng Gu, Junying Ma, Jianhua Ju

**Affiliations:** 1 CAS Key Laboratory of Tropical Marine Bio-Resources and Ecology, RNAM Center for Marine Microbiology, Guangdong Key Laboratory of Marine Materia Medica, South China Sea Institute of Oceanology, Chinese Academy of Sciences, Guangzhou 510301, China; 2 University of Chinese Academy of Sciences, Beijing 110039, China; 3 Southern Marine Science and Engineering Guangdong Laboratory (Guangzhou), Guangzhou 511458, China; 4 Guangdong Provincial Key Laboratory of Medical Molecular Diagnostics, Institute of Laboratory Medicine, Guangdong Medical University, Dongguan 523808, Chinahuazhang@gdmu.edu.cn (H.Z.); 5 Syngenta Jealott’s Hill International Research Centre, Bracknell RG42 6EY, Berkshire, UK

**Keywords:** OSMAC, biosynthetic gene clusters (BGCs), angucyclines, streptophenazines, tumor cytotoxic activity

## Abstract

The one strain many compounds (OSMAC) strategy is an effective method for activating silent gene clusters by cultivating microorganisms under various conditions. The whole genome sequence of the marine-derived strain* Streptomyces globisporus* SCSIO LCY30 revealed that it contains 30 biosynthetic gene clusters (BGCs). By using the OSMAC strategy, three types of secondary metabolites were activated and identified, including three angucyclines, mayamycin A (**1**), mayamycin B (**2**), and rabolemycin (**3**); two streptophenazines (streptophenazin O (**4**) and M (**5**)); and a macrolide dimeric dinactin (**6**), respectively. The biosynthetic pathways of the secondary metabolites in these three families were proposed based on the gene function prediction and structural information. The bioactivity assays showed that angucycline compounds **1**–**3** exhibited potent antitumor activities against 11 human cancer cell lines and antibacterial activities against a series of Gram-positive bacteria. Mayamycin (**1**) selectively exhibited potent cytotoxicity activity against triple-negative breast cancer (TNBC) cell lines such as MDA-MB-231, MDA-MB-468, and Bt-549, with IC_50_ values of 0.60–2.22 μM.

## 1. Introduction

Marine-derived *Streptomyces* are an important source of secondary metabolites with biological activities and diverse chemical structures due to their unique habitats and metabolic pathways [1,2,3]. Although genome sequencing and bioinformatic analyses revealed that there are about 20–40 biosynthetic gene clusters (BGCs) encoding the secondary metabolism in each *Streptomyces* genome, most of the gene clusters are “silent” or have low expression under the traditional culture conditions [4]. The OSMAC strategy has proven to be an effective method for discovering new cryptic natural products in microorganisms through modification of the growth conditions without genetic manipulation of the producing strains. The OSMAC strategy was first developed by Zeeck’s group in 2002 [5] and included methods such as changing nutritional conditions, trace elements, and physical parameters (i.e., pH and temperature), adding chemical composition (i.e., inhibitors and communication molecules), and co-culture [5,6,7]. By using the OSMAC strategy, Zeeck’s group discovered more than 100 secondary metabolites representing 25 structural types from 6 microorganisms [5]. In recent years, the OSMAC strategy has attracted large number of scholars, who apply it to the study of microbial secondary metabolites discovery because it has the advantages of easy operation, short cycle, low cost, and remarkable results. For instance, three rare anti-infective 22-membered macrolactone polyketides were isolated from *Streptomyces* sp. strain C34 using a range of cultivation media [8]. Twelve new cytochalasins were discovered from the fungus *Phomopsis* sp. QYM-13 by changing the media nutrients [9]; a coculture of *Streptomyces* sp. with *Pandoraea* sp. produced antitumor 6-deoxy-α-l-talopyranose-bearing aromatic metabolites [10]; and so on.

While searching for new secondary metabolites of anti-infective and anti-tumor bioactivities from marine actinomycetes, we encountered a marine-derived actinomycete *Streptomyces globisporus* SCSIO LCY30. By using the OSMAC strategy, three angucyclines mayamycin A-B (**1**–**2**) and rabelomycin (**3**), two streptophenazines (**4**–**5**), and a macrolide dimeric dinactin (**6**) were produced by *Streptomyces globisporus* SCSIO LCY30. Compounds **1**–**3** exhibited potent cytotoxicity against several human cancer cell lines and antibacterial activity against a series of Gram-positive bacteria. Herein, the whole genome sequence of *Streptomyces globisporus* SCSIO LCY30, the isolation, structure elucidation, and bioactivity of compounds **1**–**6**, and the biosynthetic pathway analysis of these secondary metabolites were reported.

## 2. Results and Discussion

### 2.1. Identification and Genome Analysis of the Strain SCSIO LCY30

**Figure 1:**
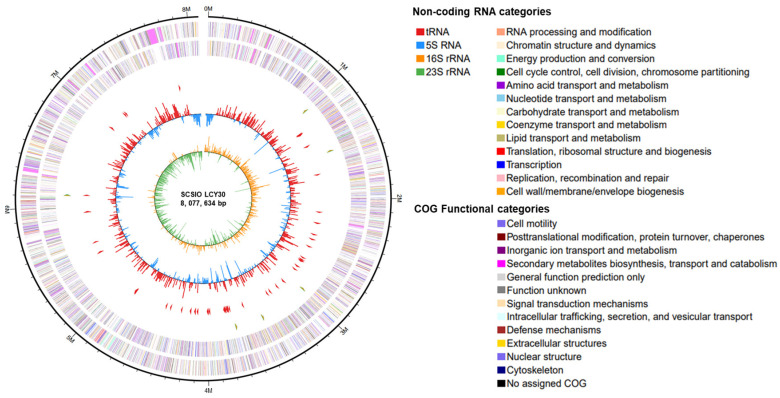
The complete genomic graph of *Streptomyces globisporus* SCSIO LCY30. These six circles (from outer to inner) represent the size of the genome, CDs on positive chains, CDs on negative chains (different colors indicate different COG functional classifications for CDS), rRNA and tRNA, GC content, and GC skew value.

SCSIO LCY30 is a marine-derived actinomycete from Weizhou Island, Guangxi Beibu Gulf, China. Analysis of the 16S rDNA and morphological characteristics suggested that SCSIO LCY30 was likely a *Streptomyces* sp. The 16S rRNA gene sequence was deposited in GenBank under accession number OR342326. The whole genome sequencing of SCSIO LCY30 was carried out by PacBio RS and Illumina sequencing technologies. The genome sequence results showed that SCSIO LCY30 has a linear chromosome with the length of 8,077,634 bp and that the average G+C content is 71.56%. The genome was predicted to contain 6990 protein-coding genes, 18 rRNA genes, and 66 tRNA genes (Figure 1).

By using autoMLST to construct a phylogenomic tree of SCSIO LCY30 in the “denovo mode” with the default settings [11], the results showed that the sequence similarities between SCSIO LCY30 and *Streptomyces globisporus* C-1027 (accession number: GCF 000261345, ANI: 97.5%, AAI: 96.77%) were the highest (Figure S1). Based on phylogenomic analysis of the whole genome sequences, the strain SCSIO LCY30 was identified as *Streptomyces globisporus*. To identify the secondary metabolite BGCs of SCSIO LCY30, the whole genome sequence was analyzed by antiSMASH version 7.1.0 online tools [12]. The analysis results revealed that there are at least 30 putative BGCs, including 3 nonribosomal peptide clusters (NRPS), 2 polyketide clusters (Type I or Type III PKS), 5 hybrid PKS-NRPS clusters, 4 terpene clusters, and several other gene clusters, such as butyrolactone, lassopeptide, ribosomal peptide (RiPP), and so on (Table S1).

### 2.2. Production of Angucyclines and Streptophenazines via OSMAC Strategies

**Figure 2:**
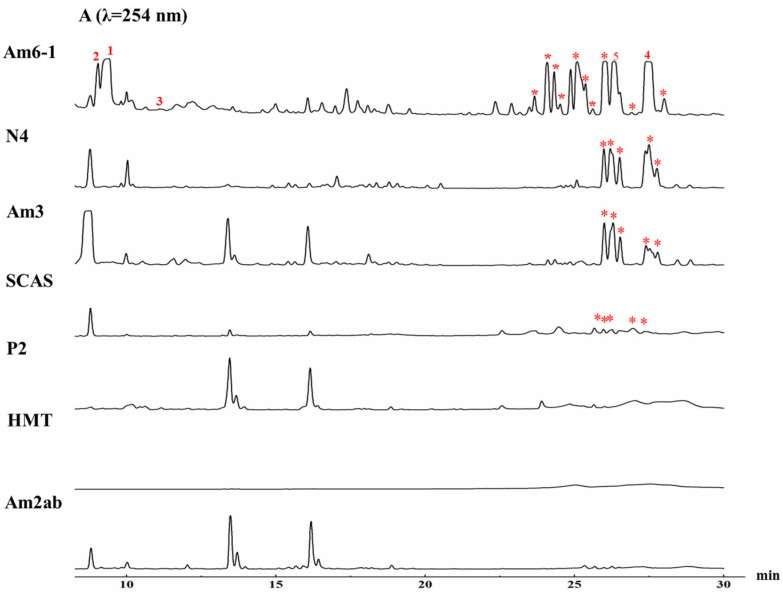
Metabolite analyses HPLC of *Streptomyces globisporus* SCSIO LCY30 fermented in seven different culture media using OSMAC strategy. (The product peaks of compounds **1**–**5** have been marked, streptophenazines product peaks have been marked with *).

In order to explore the secondary metabolites produced by SCSIO LCY30, we used conventional actinomycetes media, such as P2 [13], HMT [14], etc., to ferment the SCSIO LCY30 strain; at first, very few secondary metabolites were detected (Figure 2). However, the genome analysis of the SCSIO LCY30 strain showed that it contained 30 BGCs, which indicated that the strain had the potential to be further explored. The OSMAC strategy has proven to be an effective method for discovering new cryptic natural products, and the change in nutrient regimes is regarded as one of the effective methods in the OSMAC strategy [6]. Five other different culture media (N4 [15], Am2ab [16], Am3 [16], Am6-1 [17], and SCAS [18]) were used to ferment the SCSIO LCY30 strain, and the fermentation extracts were analyzed by HPLC. Fortunately, the UV absorption peaks of two groups, like angucyclines and streptophenazines, were mainly observed in three culture media (N4, Am3, and Am6-1) (Figure 2). The fermentations carried out in Am6-1 media by adding 2% XAD16N resins presented optimum yields. Among the 30 putative BGCs of SCSIO LCY30, cluster 20 is predicted to be a T1PKS, T2PKS, and phenazines hybrid cluster. Based on the above results and the analysis by antiSMASH, we speculated that cluster 20 might be activated due to the co-expression of adjacent gene clusters in bacteria [19,20]. 

### 2.3. Isolation and Characterization of Compounds ***1***–***6***

**Figure 3:**
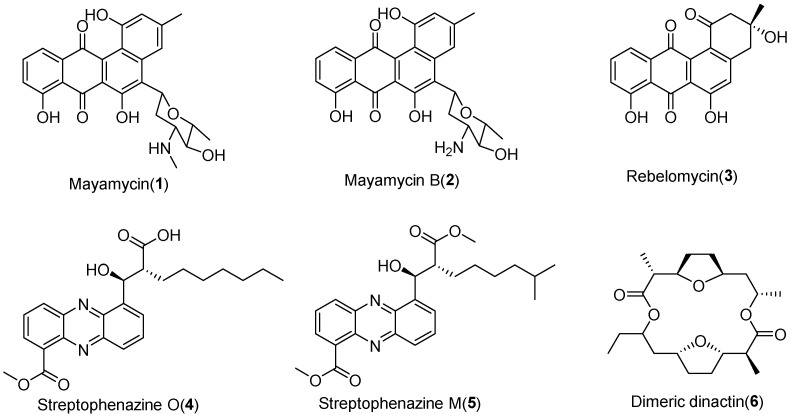
The structures of compounds **1**–**6**.

A 20-liter fermentation of SCSIO LCY30 was performed in AM6-1 media adding XAD16N resins, which resulted in the isolation and characterization of compounds **1**–**6**. HRESIMS analysis showed that the molecular formula of compounds **1** and **2** was found to be C_26_H_26_NO_7_ and C_25_H_23_NO_7_, respectively. Combined with 1D (^1^H, ^13^C) NMR, 2D (HMBC, HSQC, NOESY, and COSY) NMR spectroscopic data, and HRESIMS data, the structures of compounds **1**–**2** were identified as the angucycline compounds mayamycin (**1**) and mayamycin B (**2**), according to the literature, respectively [21,22]. The molecular formula of compound **3** was found to be C_19_H_14_O_6_ on the basis of the HRESIMS peak at *m*/*z* 339.0858 [M+H]^+^ and 361.0690 [M+Na]^+^. Compared with the reported literature, the structure of compound **3** was identified as the angucycline compound rabelomycin (**3**) based on its HRESIMS data and 1D (^1^H, ^13^C) NMR spectroscopic data [23]. The ^1^H and ^13^C NMR spectra of compound **4** showed two groups of signals with an integration ratio of 1:4; their structures were identified as streptophenazine O (**4**) and dimeric dynactin (**6**) based on the comparisons with the previous literature [24,25]. Like compound **4**, the ^1^H and ^13^C NMR spectra of compound **5** also showed two groups of signals with an integration ratio of 1:5; their structures were identified as streptophenazine M (**5**) and dimeric dynactin (**6**) [24,25]. The structures of these compounds are shown in Figure 3. 

### 2.4. Proposed Biosynthetic Pathway of Angucycline Compounds ***1***–***3***

**Figure 4:**
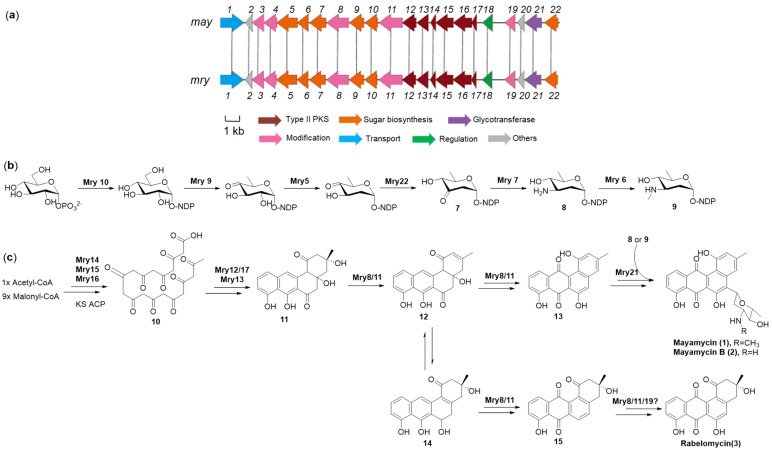
Biosynthetic gene cluster and proposed biosynthetic pathway of compounds **1**–**3**. (**a**) Genetic organization and comparison of the mayamycin biosynthetic gene cluster from *Streptomyces* sp. 120454 (*may*, upper) and *Streptomyces globisporus* SCSIO LCY30 reported in this study (*mry*, lower) using clinker. (**b**) Proposed biosynthetic pathway of sugars of compounds **1**–**2**. (**c**) Proposed biosynthetic pathway of compounds **1**–**3**.

Type II polyketide synthases are responsible for the biosynthesis of the angucycline natural products skeleton. The biosynthesis of angucycline compounds **1**–**3** also follows this paradigm. In the genome of *Streptomyces* SCSIO LCY30, only one type II polyketides gene cluster was found, which showed high similarity with the reported mayamycin BGC of *Streptomyces* sp. 120454 [22]. The putative *mry* gene cluster (accession number: OR345931) covers a 23.2 kb contiguous DNA region, including six type II PKS genes, six sugar biosynthetic genes, five modified genes, a glycotransferase gene, one transport gene, one regulatory gene, and two other genes (Figure 4a, Table S2). 

Based on the general biosynthesis paradigm of angucyclines, the structural feature of compounds **1**–**3**, and the bioinformatic analysis of the *mry* gene cluster, we proposed the biosynthetic pathway of angucycline compounds **1**–**3** (Figure 4b,c). Firstly, type II polyketide synthases Mry16 (ketosynthase alpha, KSα), Mry15, (chain length factor, CLF), and Mry14 (acyl carrier protein, ACP) utilized an acetyl-CoA as a start unit and nine malonyl-CoAs as an extension unit to establish a polyketide chain **10**. Mry12 (homolog of cyclase/dehydrase), Mry17 (homolog of cyclase), and Mry13 (homolog of ketoreductase) were predicted to be responsible for the cyclization and ketoreduction from **10** to UWM6 (**11**), which is an important intermediate of most angucyclines [26]. Both May8 and May11 were annotated as a FAD-binding monooxygenase homolog, which plays an important role in the oxidation reaction of the intermediate formation of **12**, **13**, **14**, and **15.** However, the catalysis spatiotemporal order of these two enzymes in relation to those mediates is uncertain. According to the biosynthesis of aminosugar angolosamine, we proposed the biosynthetic pathway of aminosugar *N*-demethylangolosamine (**9**) and angolosamine (**8**) in compounds **1**–**2**, respectively. The formation of *N*-demethylangolosamine (**6**) may be catalyzed by six enzymes: Mry10 (NDP-glucose phosphate nucleotidyltransferase), Mry9 (NDP-hexose 4,6-dehydratase), Mry5 (NDP-deoxyglucose-2,3-dehydratase), Mry22 (NDP-4-keto-6-deoxyhexose reductase), Mry7 (NDP-deoxyhexose 3-aminotransferase), and Mry6 (*N*-Methyl transferase), in sequence [27]. Finally, under the catalyzation of Mry21 (*C*-glycosyltransferase), mayamycin and mayamycin B were formed by using either **9** or **8** as aminosugar donors, respectively. The hydroxylation catalyzed by a monooxygenase (Mry8/11/19) at C-6 position of tetrangomycin (**15**) yielded rabelomycin (**3**).

### 2.5. Proposed Biosynthetic Pathway of Streptophenazines O and M (***4***–***5***)

**Figure 5:**
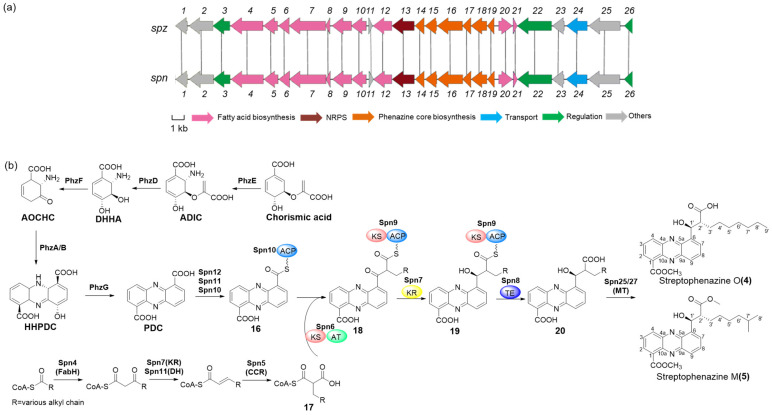
Biosynthetic gene cluster and proposed biosynthetic pathway of streptophenazines O and M (**4**–**5**). (**a**) Genetic organization and comparison of the streptophenazines biosynthetic gene cluster from *Streptomyces* sp. CNB-091 (*spz*, upper) and *Streptomyces globisporus* SCSIO LCY30 reported in this study (*spn*, lower) using clinker. (**b**) Proposed biosynthetic pathway of streptophenazines O and M (**4**–**5**).

Streptophenazines are 1,6-disubstituted phenazines with a long alkyl chain at the C-6 position. The biosynthetic gene cluster of streptophenazines was identified in *Streptomyces* sp. CNB-091 [28]. In the BGCs of *Streptomyces globisporus* SCSIO LCY30, cluster 20 is a T1PKS, T2PKS, and phenazines hybrid gene cluster. In the downstream of cluster 20 in *Streptomyces* sp. LCY30, a series of genes showed high similarity to those of the streptophenazine BGCs in *Streptomyces* sp. CNB-091 (Figure 5a and Table S3); we named it as an *spn* gene cluster (accession number: OR345932). The biosynthesis of the streptophenazines was divided into two parts: the phenazine skeleton structure synthesized by a set of contiguous phenazine core genes (*spn16-21*) and the alkyl chain at the C-6 position of streptophenazines synthesized by type I PKS genes (*spn4-11*). We proposed the biosynthetic pathway of streptophenazines O and M (**4**–**5**) produced in *Streptomyces globisporus* SCSIO LCY30 as follows (Figure 5b): phenazine-1,6-dicarboxylic acid (PDC) displays the ability to form a series of high-activity phenazine derivatives, which is also a key start unit for the biosynthesis of streptophenazines. Chorismic acid derived from the shikimate pathway in bacteria serves as a common branching point of PDC. The formation of 2-amino-4-deoxychorismic acid (ADIC) from chorismic acid is catalyzed by Spn18 (PhzE), a homolog of anthranilate synthase. Spn19 (PhzE) is predicted to be the homolog of isochorismatase, which could cleave the ADIC to trans-2,3-dihydro-3-hydroxyanthranilic acid (DHHA) [29]. Then, under the catalyzation of Spn19 (PhzE), the homolog of 2,3-Dihydro-3-hydroxylanthranilate isomerase, 6-amino-5-oxocyclohex-2-ene-1-carboxylic acid (AOCHC), is formed [30]. Spz21 (phenazine biosynthesis protein, PhzA/B) could catalyze the formation of hexahydrophenazine-1,6-dicarboxylic acid (HHPDC). The homolog of pyridoxamine 5′-phosphate oxidase, Spz16 (PhzG), catalyzes HHPDC to undergo a two-step oxidation reaction to generate PDC [29]. 

One of the most interesting aspects in the biosynthesis of streptophenazines is the PKS extension at the C-6 position. It was deduced that PDC served as the starter unit and that intermediate **17** derived from the fatty acid biosynthetic pathway acted as the extension unit for the PKS extension. The carbonyl group was reduced to the hydroxyl group at C-1′ by Spn7 (ketoreductase) in intermediate **19**; then, under the catalyzation of Spn8 (thioesterase), the polyketone chain was released to produce intermediate **20** [28]. Finally, streptophenazines O and M (**4**–**5**) were formed under the catalyzation of methyltransferase Spn25 or Spn27 [28]. However, the catalytic order of those two methyltransferases to the carboxyl group on the C-1 and C2’ position is unclear, and more in vivo evidence is required to identify the BGC of streptophenazines.

### 2.6. Proposed Biosynthetic Pathway of Macrolide Dimeric Dinactin (***6***)

**Figure 6:**
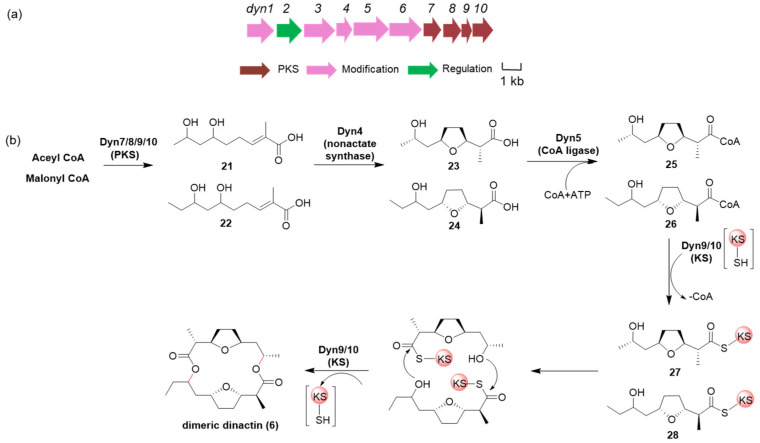
Biosynthetic gene cluster and proposed biosynthetic pathway of dimeric dynactin (**6**). (**a**) Genetic organization the dimeric dynactin (**6**) biosynthetic gene cluster from *Streptomyces globisporus* SCSIO LCY30. (**b**) Proposed biosynthetic pathway of dimeric dynactin (**6**).

Considering the structural similarity between dinactin and nonactin, we speculated the biosynthetic pathway of dimeric dinactin (**6**) based on the biosynthetic characteristics of nonactin. (Figure 6) The structure of dimeric dynactin is composed of nonactic acid (**23**) and homononactic acid (**24**). The biosynthetic gene cluster of nonactin (*non*) in *Streptomyces griseus* DSM40695 revealed a novel type of PKS for polyketide biosynthesis that consists of five type II ketoacyl synthases (KSs) and four ketoreductases (KRs) but lacks acyl carrier protein (ACP) [31]. Using local Blast, a gene cluster similar to a non-gene cluster was found in the SCSIO LCY30 genome; we named it as a *din* gene cluster (accession number: OR351017), which covers a 12.0 kb contiguous DNA region, including ten open reading frames (ORFs) (Table S4). Acetyl CoA or Malonyl CoA acts as a start unit for the biosynthesis of the polyketide chain **21** and **22** under the catalysis of KS and KR [32]. Then, it is proposed that the furan cyclic formation is accomplished by the NonS (enoyl-CoA hydratase) homolog Dyn4. Dyn5 (homolog of nonactic acid: CoASH ligase, NonL) is responsible for the transformation of **23/**(**24**) to CoA esters **25/**(**26**) [33,34]. Finally, the condensation between the -OH group of the distal nonactyl unit of **27** and the acyl-S-KS carbonyl group of **28** occurred, and the -OH group of the distal nonactyl unit of **28** and the acyl-S-KS carbonyl group of **27** also occurred to form dimeric dinactin (**6**) [34]. Interestingly, this C-O bond condensation dimeric dinactin (**6**) might be catalyzed by the ketoacyl synthases Dyn10 (NonK homolog) and Dyn9 (NonJ homolog) in nonactin biosynthesis [34]. However, the specific biosynthetic pathway of dimeric dynactin (**6**) and the catalytic mechanism of the enzyme need to be confirmed by further experimental data. 

### 2.7. GNPS Molecular Network Analysis of Streptophenazines

**Figure 7:**
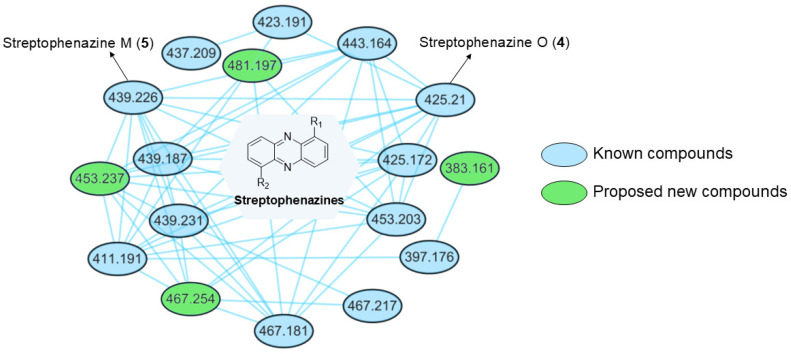
Streptophenazines cluster of nodes from the GNPS molecular network (related to Figures S2 and S3 and Table S6).

Due to the instability of streptophenazines when mixed with compound **6** (UV absorption below 210 nm), we had only previously identified two streptophenazine structures (**4**–**5**) from SCSIO LCY30. To explore the possible streptophenazine analogues that might be neglected in the initial visual analysis, the crude extract of *Streptomyces globisporus* SCSIO LCY30 was fractionated by positive ion mode LC-MS/MS; then, a GNPS molecular network was generated using the tandem mass spectra of the two isolated streptophenazines (**4**–**5**) as an anchor. The network identified 17 nodes corresponding to streptophenazine-type compounds (Figure 7 and Table S6). Compared with the structures of the streptophenazine compounds we reviewed in recent years (Figure S3) [24,28,35,36,37,38,39], the possible structures of the compounds corresponding to these nodes were speculated based on molecular weight and mass spectrum cleavage (Table S5). In the molecular network of streptophenazines, two nodes were identified as streptophenazin M (**5**) (*m*/*z* = 439.226) and O (**4**) (*m*/*z* = 425.21) in this study; the molecular weights of these 10 nodes highlighted in green were consistent with those reported for the streptophenazine compounds. In addition, four nodes marked in blue might be new compounds due to there being no matching streptophenazines compounds found for these molecular weights, and we proposed the possible structure according to the structure feature of streptophenazines (Table S6).

The above data showed that the SCSIO LCY30 strain had great potential to produce streptophenazines. In recent years, based on the wide range of biological properties and medical applications of phenazines, the synthesis, structural modification, and structure–activity relationship of phenazines have been studied extensively [40,41,42], while the biological activity of streptophenazine compounds has rarely been reported to date. Among the streptophenazine compounds that have been reported, (-)-Streptophenazine B showed moderate activity against methicillin-resistant *Staphylococcus aureus*, with an MIC value of 4.2 μg/mL [24]. Streptophenazines C and H showed moderate inhibitory activity against *Bacillus subtilis* (MIC value of 15.6 µg/mL), and streptophenazine C was also active against *Staphylococcus lentus* (MIC value of 46.9 µg/mL) [35]. And other biological activities of streptophenazine-type compounds could be explored in the future. 

### 2.8. Antibacterial Activity of Compounds ***1***–***3***

As high amounts of compounds **1**–**3** were available**,** the antibacterial activities of angucycline compounds **1**–**3** against several Gram-positive bacteria were first tested. The minimum inhibitory concentration (MIC) values are shown in Table 1. The results showed that compounds **1**–**3** exhibited inhibitory activity against many Gram-positive bacteria, such as *Staphylococcus aureus*, *Enterococcus faecium*, *Enterococcus faecalis*, *Enterococcus gallinarum*, *Bacillus subtilis*, and *methicillin-resistant Staphylococcus aureus (MRSA)*, as summarized in Table 1. It is worth noting that compounds **1**–**3** exhibited potent antibacterial activity against *Micrococcus luteus* ML01; the MIC values were 1 μg/mL, 1 μg/mL, and 2 μg/mL, respectively.

### 2.9. Tumor Cytotoxic Activity of Compounds ***1***–***3***

Given that angucycline compounds have potent antitumor activities, the half inhibitory concentration of angucycline compounds **1**–**3** against fourteen different human cell lines were then tested. The IC_50_ values are shown in Table 2. Compounds **1**–**3** also showed potent in vitro cytotoxicity against fourteen human cell lines; the IC_50_ values were in a range of 0.60~5.98 μM, 2.05~7.16 μM, and 1.57~16.13 μM, respectively. Notably, the tumor cytotoxic activities against eight human cancer cell lines of mayamycin (**1**) was superior to that of mayamycin B (**2**), suggesting that the *N*-methyl group is important for its antitumor activity. In addition, the cytotoxicity of rabelomycin (**3**) against the colon cancer cell line SW480 (IC_50_ = 1.57 μM) was lower than that against the normal intestinal epithelial cell line NCM460 (IC_50_ = 7.05 μM); the selective index (SI) was 4.49, which is expected to be developed as an anti-colorectal cancer drug lead. 

Triple-negative breast cancer (TNBC), once known as “the most malignant breast cancer”, is a great challenge for clinical management due to the lack of therapeutic targets and limited therapeutic drugs. The anti-TNBC activities of compounds **1**–**3** have not been reported. In this study, we also tested the inhibitory activity of compounds **1**–**3** against TNBC cell lines. It is worth noting that compounds **1**–**3** exhibited potent in vitro cytotoxicity against TNBC cell lines such as MDA-MB-231, MDA-MB-468, and Bt-549; the IC_50_ values were in a range of 0.60~2.22 μM, 3.01~6.08 μM, and 2.18~8.67 μM, respectively. Among compounds **1**–**3**, mayamycin (**1**) showed the best cytotoxicity activity against the TNBC cell lines MDA-MB-231, MDA-MB-468, and Bt-549 (IC_50_ = 0.60 μM, 2.22 μM, and 1.88 μM); the selective indexes were 9.97, 2.69, and 3.18, which indicated that the toxicity and side effects of mayamycin (**1**) on normal breast cells were relatively weak and that it has the potential to become a drug lead compound for the treatment of TNBC. 

## 3. Materials and Methods

### 3.1. General Experiment Procedure

HRESIMS and HRESIMS/MS spectra were measured by a MaXis 4G UHR59 TOFMS spectrometer. All 1D and 2D NMR spectra were acquired by a Bruker Ascend 700 spectrometer (Bruker Company, Karlsruhe, Germany) with TMS as the internal standard (Sigma-Aldrich Inc., Vienna, Austria) Silica gel (100−200 mesh and 200−300 mesh; Yantai Jiangyou Silica Gel Development company, Yantai, China) was used for column chromatography. Analytic HPLC was performed by an Agilent 1260 HPLC system equipped with a G1311C isocratic pump and an Agilent G1315D diode array detector (DAD), using a reversed-phase column Basic C18 120A (Agilent company, 4.6 × 250 mm, 5 μm, Santa Clara, CA, USA). Semipreparative HPLC was performed by an Agilent 1260 HPLC system equipped with a 1110 isocratic pump and a 1430 DAD detector, using an ODS-A column (YMC company, 10 mm × 250 mm, 5 μm, Kyoto, Japan).

### 3.2. Producing Strain and Genome Scanning

The SCSIO LCY30 strain was isolated from Weizhou Island, Guangxi Beibu Gulf, China. The genome sequencing of SCSIO LCY30 was performed by 2nd Illumina sequencing technologies and 3rd PacBio RS platforms in Shanghai BIOZERON biotechnology CO., LTD (Shanghai, China).

### 3.3. Phylogenetic Construction and Bioinformatic Analysis

The phylogenomic tree of the SCSIO LCY30 whole genome sequences was completed by autoMLST in the “denovo mode” with default settings [11], and the phylogenomic tree was beautified by iTOL software (version 6.7.2) [43]. The average amino acid identity of the different genomes was determined by ANI calculator [44]. The secondary metabolite BGCs of SCSIO LCY30 were identified and analyzed using the online software antiSMASH version 7.1.0 [12]. The sequences alignment of the gene clusters was completed by clinker software (https://cagecat.bioinformatics.nl/tools/clinker, accessed on 9 May 2023). 

### 3.4. Fermentation Conditions

*Streptomyces globisporus* SCSIO LCY30 was inoculated to MS agar plates (mannitol 20 g/L, soybean powder 20 g/L, agar 20 g/L, pH 7.2–7.4) and incubated at 28 °C for 5–7 days. Seven different media were used to culture the SCSIO LCY30 strain to modify the fermentation conditions, and the media formulations are as follows: N4 medium (soluble starch 15 g/L, fish peptone 8 g/L, bacteria peptone 5 g/L, glycerin 8 g/L, KBr 0.2 g/L, sea salt 30 g/L, CaCO_3_ 2 g/L, pH 7.2–7.4) [15]; P2 medium (malt extract 10 g/L, glucose 4 g/L, yeast extract 5 g/L, sea salt 30 g/L, CaCO_3_ 2 g/L, pH 7.2–7.4) [13]; Am2ab medium (soluble starch 5 g/L, glucose 20 g/L, yeast extract 2 g/L, bacteria peptone 2 g/L, soybean powder 5 g/L, KH_2_PO_4_ 0.5 g/L, MgSO_4_·7H_2_O 0.5 g/L, NaCl 4 g/L, sea salt 30 g/L, CaCO_3_ 2 g/L, pH 7.2–7.4) [16]; HMT medium (fish meal 10 g/L, yeast extract 5 g/L, glycerin 20 g/L, CaCO_3_ 5 g/L, pH 7.2–7.4) [14]; Am3 medium (soluble starch 10 g/L, soybean powder 5 g/L, glycerin 10 g/L, pepotne 15 g/L sea salt 30 g/L, CaCO_3_ 2 g/L, pH 7.2–7.4) [17]; Am6-1 (starch 20 g/L, glycerine 10 g/L, yeast extract 5 g/L, sea salt 30 g/L, and CaCO_3_ 2 g/L, pH 7.2–7.4) [17]; and SCAS medium (soluble starch 40 g/L, casamino acid 5 g/L, K_2_HPO_4_ 0.5 g/L, MgSO_4_·7H_2_O 0.5 g/L, FeSO_4_·7H_2_O 0.01 g/L, CaCO_3_ 2 g/L, pH 7.2–7.4) [18]. The mycelium was transferred into the 250 mL flasks containing 50 mL of Am6-1 medium and then incubated on rotary shakers (28 °C, 200 rpm) for 36 h. After 36 h of growth, each of the seed flasks, respectively, was transferred to two 1 L flasks that contained 200 mL of Am6-1 medium with the addition of 2% XAD16N resins, and then incubated on rotary shakers (28 °C, 200 rpm) for 7 days. 

### 3.5. Purification and Characterization of Compounds

About 20 L of fermentation culture was obtained, and the XAD16N resins were separated from the fermentation broth through a sieve. The fermentation broth was centrifuged at 3500 rpm for 15 min to separate the supernatant and the mycelia. The mycelia were extracted by acetone, and the XAD16N resins were extracted using EtOH by ultrasonication three times. The extract solution was combined, and 24 g crude material was obtained by vacuum concentration. The crude material was subjected to a silica gel CC using gradient elution with CHCl_3_ and MeOH mixtures (100:0, 98:2, 96:4, 94:6, 92:8, 90:10, 80:20, 70:30, 60:40, 50:50) to give ten fractions (Fr. 1–Fr. 10). Fr. 1-4 were combined and then subjected to a silica gel CC using gradient elution with petroleum ether and ethyl acetate mixtures (100:0, 9:1, 8:2, 7:3, 6:4, 5:5, 4:6, 3:7. 2:8, 1:9, 0:100) to give eleven fractions (Fr. 4.1–Fr. 4.11). 

Fr. 7 was separated by semipreparative HPLC (MeCN−H_2_O, 27:73) to yield compound **1** (75.1 mg, *t_R_* = 14.5 min) and compound **2** (25.6 mg, *t_R_* = 20.5 min). Fr. 4.10 was separated by semipreparative HPLC (MeCN–H_2_O, 35:65) to yield compound **3** (7.5 mg, *t_R_* = 15.5 min). Fr. 4.5 was separated by semipreparative HPLC (MeCN−H_2_O, 0–40 min, 30:70–85:15) to yield the mixture of compound **4** and compound **6** (7.5 mg, *t_R_* = 15.5 min). Fr. 4.6 was separated by semipreparative HPLC (MeCN–H_2_O, 0–40 min, 30:70–85:15) to yield the mixture of compound **5** and compound **6** (12.5 mg, *t_R_* = 18.5 min).

### 3.6. Production Analysis by HPLC

The metabolites of the gene disruption mutant strains were analyzed using a reversed-phase column Basic C18 120A (Agilent, 4.6 × 250 mm, 5 μm) with a DAD detector using the solvent system (phase A, 15% CH_3_CN + 0.1% HAc; phase B, 85% CH_3_CN + 0.1% HAc): 0–20 min 0−80% phase B; 20–21.5 min 80–100% phase B; 21.5−27 min 100% phase B; 27–27.1 min 100−0% phase B; 27.1–30 min 0% phase B at a flow rate of 1 mL/min. 

### 3.7. Antibacterial Activity Assays

An antibacterial activity test of compounds **1**–**3** was conducted according to a standard protocol provided by the Clinical and Laboratory Standards Institute (CLSI) [45,46]. Sixteen bacterial strains were used, including *Staphylococcus aureus* ATCC 29213, *Staphylococcus aureus* 16339, *Staphylococcus aureus* 1862, *Staphylococcus aureus* 3090, *Staphylococcus aureus* 991, *Staphylococcus aureus* 669, *Staphylococcus aureus* 745324, *Staphylococcus aureus* 16162, *Staphylococcus aureus (cfr)* GDE4P037P, *Enterococcus faecalis* ATCC 29212, *Enterococcus faecalis* 36950, *Micrococcus luteus* ML01, MRSA, *Bacillus subtilis* BS01, *Enterococcus gallinarum* 5F52C, and *Enterococcus faecium* 36711. Compounds **1**–**3** were dissolved in DMSO at a concentration of 3.2 mg/mL, and vancomycin and ampicilin were used as antibacterial control agents. After incubation at 37 °C for 16–18 h, the microdilution instrument was used to detect the minimum concentration of each tested compound that completely inhibited bacterial growth in the microdilution hole. All the assays were carried out in triplicate.

### 3.8. Cytotoxic Activity Assays

The cell growth inhibitory activities of compounds **1**–**3** against fourteen different human cell lines (human liver cell line LX-2; hepatocellular cancer line HEPG2; human intestinal epithelial cell line NCM460; colorectal cancer cell lines HCT116 and SW480; normal human breast cell line MCF-10A; breast cancer cell line MCF7; triple-negative breast cancer cell lines MDA-MB-231, MDA-MB-468, and Bt-549; human umbilical vein endothelial cell line; lung cancer cell line A549; cervical cancer cell line Hela; and cholangiocarcinoma cell line RBE) were tested according to the previously published methods [47]. SPSS software version 22.0 was used to test the IC_50_ values. Cisplatin and adriamycin were used as positive control agents, and all the experiments were carried out in triplicate.

### 3.9. Construction of GNPS (Global Natural Products Social) Molecular Network

The molecular networking analysis was accomplished using online GNPS software [48] (https://gnps.ucsd.edu/ProteoSAFe/static/gnps-splash.jsp, accessed on 24 March 2023). The output molecular network was edited and visualized using Cytoscape_v3.9.1 software.

## 4. Conclusions

In summary, the whole genome sequence of marine-derived *Streptomyces globisporus* SCSIO LCY30 was acquired, and the bioinformatic analysis showed at least 30 BGCs encoding various secondary metabolites, among which cluster 20 contains an angucycline biosynthesis gene cluster and a phenazine biosynthesis gene cluster. Three angucyclines, including mayamycin (**1**), mayamycin B (**2**), rabelomycin (**3**), two streptophenazines (streptophenazines O (**4**) and M (**5**)), and macrolide dimeric dinactin (**6**), were isolated from the marine-derived *Streptomyces globisporus* SCSIO LCY30 by using the OSMAC strategy under different culture conditions. The antibacterial bioassay results showed that compounds **1**–**3** showed potent bioactivity against *Micrococcus luteus*, with MIC values of 1.0 to 2.0 μg/mL. Furthermore, compounds **1**–**3** showed potent in vitro cytotoxicity against 11 human cancer cell lines. Mayamycin (**1**) selectively exhibited potent cytotoxicity activity against TNBC cell lines such as MDA-MB-231, MDA-MB-468, and Bt-549 (IC_50_ = 0.60 μM, 2.22 μM, and 1.88 μM); the selective indexes were 9.97, 2.69, and 3.18, respectively, which indicated that mayamycin (**1**) is a promising drug lead compound for the treatment of TNBC. Rabelomycin (**3**) selectively exhibited potent cytotoxicity against colon cancer cell line SW480 (IC_50_ = 1.57 μM), with the selective index of 4.49, which is expected to be developed as an anti-colorectal cancer drug lead. In addition, the biosynthetic pathways of compounds **1**–**6** were determined by in vivo and in silico analysis, which provides ideas for the subsequent biosynthetic mechanism studies of these compounds. This study not only serves as a new strain resource for antitumor drug development but also lays a foundation for the mining of the rest of the other biosynthetic gene clusters that encode natural products.

## Figures and Tables

**Table 1:** Antibacterial activity of compounds **1**–**3** against bacterial strains (MIC, μg/mL).

Bacterial Strains	MIC (μg/mL) of Standard		MIC (μg/mL) of Compounds 1–3		
	Vancomycin	Ampicillin	Mayamycin (1)	Mayamycin B (2)	Rabelomycin (3)
*Staphylococcus aureus* ATCC 29213	1	1	16	16	8
*Staphylococcus aureus* 16339	1	64	16	16	8
*Staphylococcus aureus* 1862	1	4	16	16	8
*Staphylococcus aureus* 3090	0.5	16	16	8	8
*Staphylococcus aureus* 991	0.5	4	16	16	16
*Staphylococcus aureus* 669	1	8	16	16	16
*Staphylococcus aureus* 745324	1	4	16	8	8
*Staphylococcus aureus* 16162	0.5	16	16	16	8
*Staphylococcus aureus* (cfr) GDE4P037P	1	16	8	8	4
*Staphylococcus simulans* AKA1	0.5	>64	32	64	16
*Enterococcus faecalis* ATCC 29212	2	1	32	16	8
*Enterococcus faecalis* 36950	0.5	>64	8	8	8
*Micrococcus luteus* ML01	0.13	0.13	1	1	2
MRSA	0.5	32	8	8	8
*Bacillus subtilis* BS01	0.13	0.13	8	8	4
*Enterococcus gallinarum* 5F52C	4	8	32	32	32
*Enterococcus faecium* 36711	>64	>64	16	32	32

Vancomycin and ampicillin were used as positive controls.

**Table 2:** Antitumor activity of compounds **1–3** against human cancer cell line (IC_50_, μM).

Human Cell Line	IC_50_(μM) of the Standard		IC_50_(μM) of Compounds 1–3		
	Cisplatin	Adriamycin	Mayamycin (1)	Mayamycin B (2)	Rabelomycin (3)
LX-2	12.13	1.36	2.31	5.33	13.42
HEPG2	11.02	15.47	2.12	6.00	8.64
NCM460	3.17	3.89	2.64	7.16	7.05
HCT116	4.83	1.54	1.08	2.05	4.72
SW480	1.82	17.49	1.05	2.87	1.57
MCF-10A	6.43	3.72	5.98	5.21	11.31
MCF7	4.56	3.05	2.10	4.15	4.48
MDA-MB-231	15.66	4.04	0.60	3.01	8.67
MDA-MB-468	8.35	3.16	2.22	6.08	2.18
Bt-549	7.54	3.75	1.88	3.87	7.85
HUVEC	7.70	11.97	1.68	4.41	10.92
A549	9.31	1.51	1.65	5.08	5.97
Hela	7.62	7.02	0.91	2.80	16.13
RBE	13.48	4.26	1.05	4.06	4.23

Cisplatin and adriamycin were used as positive controls (human liver cell line LX-2, hepatocellular cancer line HEPG2; human intestinal epithelial cell line NCM460; colorectal cancer cell lines HCT116 and SW480; normal human breast cell line MCF-10A; breast cancer cell line MCF7; triple-negative breast cancer cell lines MDA-MB-231, MDA-MB-468, and Bt-549; human umbilical vein endothelial cell line; lung cancer cell line A549; cervical cancer cell line Hela; and cholangiocarcinoma cell line RBE).

## Data Availability

The data presented in this study are available on request from the corresponding authors.

## Supplementary Materials

The following supporting information can be downloaded at: https://www.mdpi.com/article/10.3390/md22010021/s1, Figure S1: The phylogenetic tree of marine-derived SCSIO LCY30, Figure S2: Molecular network showing production of streptophenazines, related to Figure 7, Figure S3: Structures of streptophenazines, Figures S4–S25: NMR and MS data of compounds **1**–**6**, Table S1: The antiSMASH-predicted BGCs for *Streptomyces globisporus* SCSIO LCY30, Table S2: Deduced functions of *orfs* in the *mry* BGC, Table S3: Deduced functions of *orfs* in the *spn* BGC, Table S4: Deduced functions of *orfs* in the *din* BGC, Table S5: Summary of ^1^H and ^13^C NMR data for compounds **1**–**2** (*δ* in ppm), Table S6: High resolution ESI MS/MS (HRESIMS/MS), retention time, and predicted chemical formula for nodes in streptophenazine cluster from GNPS molecular network (related to Figure 3 and Figure 5).
