# Supplementary material for: OSMAC-Based Discovery and Biosynthetic Gene Clusters Analysis of Secondary Metabolites from Marine-Derived Streptomyces globisporus SCSIO LCY30
Source: Mar Drugs. 2023 Dec 28;22(1):21. doi: 10.3390/md22010021 (PMC10817512; doi:10.3390/md22010021)
Supplement: Supplementary file 1 [file marinedrugs-22-00021-s001.zip › marinedrugs-2801821-supplementary.pdf]

Supporting Information

OSMAC-Based Discovery and Biosynthetic Gene Clusters Analysis of Secondary Metabolites from Marine-Derived *Streptomyces globisporus* SCSIO LCY30

Yanqing Li [1,2,3], Naying Gong [4], Le Zhou [1,3], Zhijie Yang [1,2,3], Hua Zhang [4], Yucheng Gu [5], Junying Ma [1,2,3,*] and Jianhua Ju [1,2,3,*]

1 CAS Key Laboratory of Tropical Marine Bio-Resources and Ecology, RNAM Center for Marine Microbiology, Guangdong Key Laboratory of Marine Materia Medica, South China Sea Institute of Oceanology, Chinese Academy of Sciences, Guangzhou 510301, China
2 University of Chinese Academy of Sciences, Beijing 110039, China
3 Southern Marine Science and Engineering Guangdong Laboratory (Guangzhou), Guangzhou 511458, China
4 Guangdong Provincial Key Laboratory of Medical Molecular Diagnostics, Institute of Laboratory Medicine, Guangdong Medical University, Dongguan 523808, China; huazhang@gdmu.edu.cn (H.Z.)
5 Syngenta Jealott's Hill International Research Centre, Bracknell RG42 6EY, Berkshire, UK

* Correspondence: majunying@scsio.ac.cn (J.M.); jju@scsio.ac.cn (J.J.); Tel.: +86-20-8902-3028 (J.J.)

Table S1. The antiSMASH-predicted BGCs for *Streptomyces* sp. SCSIO LCY30

BGC	From	To	Type	Most similar known cluster	Similarity
Cluster1	111,597	131,563	terpene	2-methylisoborneol	100%
Cluster2	175,613	185,779	butyrolactone	coelimycin P1	16%
Cluster3	213,615	280,831	NRPS, terpene, NAPAA	stenothricin	13%
Cluster4	307,996	353,352	NRPS	streptobactin	100%
Cluster5	360,960	410,654	NRPS	coelichelin	81%
Cluster6	411,713	485,146	terpene, NRPS	isorenieratene	100%
Cluster7	496,705	535,481	T3PKS	herboxidiene	7%
Cluster8	1,133,865	1,152,667	terpene	steffimycin D	19%
Cluster9	1,585,298	1,595,726	ecotine	ectoine	100%
Cluster10	2,213,771	2,286,646	NRPS, NRPS-like, T3PKS	viguiepinol	73%
Cluster11	2,608,509	2,638,799	lanthipeptide-class-ii, lanthipeptide-class-iii		
Cluster12	2,693,162	2,703,155	siderophore	desferrioxamin B	100%
Cluster13	3,096,186	3,128,710	thiopeptide, LAP		
Cluster14	3,806,311	3,829,364	lanthipeptide-class-i		
Cluster15	4,456,955	4,479,652	lassopeptide	keywimysin	100%
Cluster16	4,612,207	4,621,963	butyrolactone	kedarcidin	1%
Cluster17	5,195,441	5,236,957	T1PKS		
Cluster18	5,332,444	5,355,164	lanthipeptide-class-iii	AmfS	100%
Cluster19	5,684,214	5,703,231	terpene		
Cluster20	5,724,303	5,815,609	T2PKS, T1PKS, PKS-like, phenazine	streptophenazines	100%
Cluster21	6,163,107	6,177,812	siderophore	ficellomycin	3%
Cluster22	6,251,244	6,323,647	NRPS, NRPS-like	daptomycin	14%
Cluster23	6,504,656	6,513,179	RiPP-like		
Cluster24	7,096,294	7,122,422	terpene	hopene	69%
Cluster25	7,176,618	7,225,311	NRPS, T1PKS	SGR PTMs	100%
Cluster26	7,316,149	7,357,492	butyrolactone, terpene, lanthipeptide-class-i	isorenieratene	87%
Cluster27	7,392,554	7,403,351	RiPP-like	tetronasin	3%
Cluster28	7,608,211	7,618,690	melanin	melanin	100%
Cluster29	7,657,838	7,821,877	T3PKS, NRPS, NRPS-like	herboxidiene	11%
Cluster30	7,872,061	7,924,327	T1PKS, NRPS	kanamycin	2%

Table S2. Deduced functions of *orfs* in the *mry* BGC

orfs	Size (aa)	Proposed function	ID/SI	Protein homologue	origion
mry1	543	MFS transporter	99/100	WP_260821961.1	*Streptomyces* sp. CS-7
mry2	158	nuclear transport factor 2 family protein	100/100	WP_260821960.1	*Streptomyces* sp. CS-7
mry3	266	4'-phosphopantetheinyl transferase superfamily protein	99/100	WP_260821958.1	*Streptomyces* sp. CS-7
mry4	295	alpha/beta hydrolase	99/100	WP_260821956.1	*Streptomyces* sp. CS-7
mry5	470	NDP-hexose 2,3-dehydratase family protein	100/100	WP_260821954.1	*Streptomyces* sp. CS-7
mry6	253	class I SAM-dependent methyltransferase	100/100	WP_260821952.1	*Streptomyces* sp. CS-7
mry7	373	aminotransferase	99/99	WP_086672685.1	*Streptomyces* sp. TSRI0445
mry8	504	FAD-dependent monooxygenase	99/99	WP_260821949.1	*Streptomyces* sp. CS-7
mry9	331	dTDP-glucose 4,6-dehydratase	98/99	NED04942.1	*Streptomyces* sp. SID6648
mry10	288	glucose-1-phosphate thymidylyltransferase RfbA	98/99	NED04943.1	*Streptomyces* sp. SID6648
mry11	536	FAD-dependent monooxygenase	99/100	WP_260821947.1	*Streptomyces* sp. CS-7
mry12	313	aromatase/cyclase	99/100	WP_260821945.1	*Streptomyces* sp. CS-7
mry13	262	3-oxoacyl-ACP reductase	98/100	AVO00812.1	*Streptomyces* sp.
mry14	117	acyl carrier protein	98/98	AVO00813.1	*Streptomyces* sp.
mry15	404	ketosynthase chain-length factor	99/99	WP_260821940.1	*Streptomyces* sp. CS-7
mry16	423	beta-ketoacyl-[acyl-carrier-protein] synthase	99/99	WP_073774800.1	*Streptomyces* sp. CS-7
mry17	109	TcmI family type II polyketide cyclase	99/100	WP_073774798.1	*Streptomyces* sp. CS-7
mry18	224	response regulator transcription factor	97/98	AVO00817.1	*Streptomyces* sp.
mry19	235	monooxygenase	45/60	MBT2507467.1	*Streptomyces sp.* ISL-98
mry20	159	hypothetical protein	97/98	WP_086670719.1	*Streptomyces* sp.
mry21	379	glycosyltransferase	100/100	WP_260821935.1	*Streptomyces* sp. CS-7
mry22	308	NAD-dependent epimerase/dehydratase	100/100	WP_260821933.1	*Streptomyces* sp. CS-7

Table S3. Deduced functions of *orfs* in the *spn* BGC

orfs	Size	Proposed function	ID/SI	Protein homologue	origion
spn1	329	cytochrome d ubiquinol oxidase subunit II	99/100	WP_073774786.1	*Streptomyces*
spn2	439	cytochrome ubiquinol oxidase subunit I	99/99	WP_260821929.1	*Streptomyces* sp. CS-7
spn3	293	LysR substrate-binding domain-containing protein	100/100	WP_073774781.1	*Streptomyces*
spn4	598	fatty acyl-AMP ligase	100/100	WP_260821926.1	*Streptomyces* sp. CS-7
spn5	416	crotonyl-CoA carboxylase/reductase	100/100	WP_260821924.1	*Streptomyces* sp. CS-7
spn6	800	type I PKs	100/100	WP_260821922.1	*Streptomyces* sp. CS-7
spn7	351	aldo/keto reductase	100/100	WP_260821920.1	*Streptomyces* sp. CS-7
spn8	264	alpha/beta fold hydrolase	97/97	WP_086670711.1	*Streptomyces*
spn9	913	type I PKs	99/100	WP_260821917.1	*Streptomyces* sp. CS-7
spn10	109	phosphopantetheine-binding protein	100/100	WP_073774770.1	*Streptomyces*
spn11	477	aldehyde dehydrogenase family protein	100/100	WP_260821914.1	*Streptomyces* sp. CS-7
spn12	365	phenazine antibiotic biosynthesis protein	98/99	WP_079198636.1	*Streptomyces*
spn13	110	hypothetical protein	96/99	WP_181852409.1	*Streptomyces*
spn14	455	FAD-binding protein	99/99	WP_260821909.1	*Streptomyces* sp. CS-7
spn15	545	amino acid adenylation domain-containing protein	99/99	WP_260821907.1	*Streptomyces* sp. CS-7
spn16	220	phenazine biosynthesis FMN-dependent oxidase (PhzG)	99/100	WP_260821905.1	*Streptomyces* sp. CS-7
spn17	283	phenazine biosynthesis isomerase (PhzF)	99/99	WP_086670700.1	*Streptomyces albovinaceus*
spn18	627	anthranilate synthase family protein (PhzE)	99/100	WP_260821902.1	*Streptomyces* sp. CS-7
spn19	207	isochorismatase family protein (PhzD)	99/99	WP_073774760.1	*Streptomyces*
spn20	391	Phospho-2-dehydro-3-deoxyheptonate aldolase (DAHP synthetase) (PhzC)	99/99	WP_260821899.1	*Streptomyces* sp. CS-7
spn21	166	PhzA/PhzB family protein	98/98	WP_073774756.1	*Streptomyces*
spn22	344	ketoacyl-ACP synthase III family protein (FabH)	99/99	WP_073774754.1	*Streptomyces*
spn23	97	phosphopantetheine-binding protein	100/100	WP_260821896.1	*Streptomyces* sp. CS-7
spn24	846	LuxR family transcriptional regulator	99/99	WP_260821894.1	*Streptomyces* sp. CNB091
spn25	289	methyltransferase	99/97	WP_018960036.1	*Streptomyces* sp. CNB091
spn26	516	MFS_transporter	99/100	WP_018960037.1	*Streptomyces* sp. CNB091
spn27	781	FAD-dependent_oxidoreductase	97/100	WP_018960038.1	*Streptomyces* sp. CNB091
spn28	209	TetR/AcrR_family_transcriptional_regulator	97/99	WP_018960039.1	*Streptomyces* sp. CNB091

Table S4. Deduced functions of *orfs* in the *din* BGC

orfs	Size	Proposed function	ID/SI	Protein homologue	origion
dyn1	349	nonC	89/93	AAD37456.1	*Streptomyces griseus* subsp. *griseus* DSM 40695
dyn2	175	nonB	89/92	AAD37455.1	*Streptomyces griseus* subsp. *griseus* DSM 40695
dyn3	279	tetranactin resistance protein (nonR)	89/93	AAD37454.1	*Streptomyces griseus* subsp. *griseus* DSM 40695
dyn4	297	enoyl-CoA hydratase homolog (nonS)	93/94	AAC26135.1	*Streptomyces griseus* subsp. *griseus* DSM 40695
dyn5	555	putative nonactic acid:CoASH ligase (nonL)	89/92	AAC26134.1	*Streptomyces griseus* subsp. *griseus* DSM 40695
dyn6	569	putative diester hydrolase (nonD)	89/93	AAC26133.1	*Streptomyces griseus* subsp. *griseus* DSM 40695
dyn7	271	putative ketoacyl reductase (nonE)	93/94	AAD37453.1	*Streptomyces griseus* subsp. *griseus* DSM 40695
dyn8	520	putative ketoacyl reductase (nonM)	83/88	AAD37452.1	*Streptomyces griseus* subsp. *griseus* DSM 40695
dyn9	427	ketoacyl synthase II homolog (nonJ)	93/95	AAD37451.1	*Streptomyces griseus* subsp. *griseus* DSM 40695
dyn10	462	ketoacyl synthase I homolog (nonK)	92/95	AAD37450.1	*Streptomyces griseus* subsp. *griseus* DSM 40695

Table S5. Summary of ^{1}H and ^{13}C NMR data for compounds **1-2** (δ in ppm)

	Compound **1**		Compound **2**	
position	δ_C mult	δ_H (J in Hz)	δ_C mult	δ_H (J in Hz)
	$CDCl_3$, 126 MHz	$CDCl_3$, 500 MHz	MeOD, 176 MHz,	MeOD, 700 MHz
1	154.0, C		156.5, C	
2	118.6, CH	6.97, s	114.9, CH	6.67, s
3	142.5, C		143.5, C	
3-CH_3	22.3, CH_3	2.49, s	22.7, CH_3	2.42, s
4	118.1, CH	8.16, s	117.5, CH	7.97, s
4a	138.9, C		139.9, C	
5	130.0, C		126.7, C	
6	153.7, C		154.5, C	
6a	133.3, C		138.9, C	
7	193.3, C		194.3, C	
7a	114.7, C		116.3, C	
8	161.9, C		162.8, C	
9	125.2, CH	7.34, d, J = 8.3 Hz	124.8, CH	7.21, d, J =8.1 Hz
10	137.9, CH	7.73, t, J = 8.0 Hz	138.4, CH	7.51, t, J = 7.1 Hz
11	121.4, CH	7.86, t, J = 7.3 Hz	120.4, CH	7.66, t, J = 7.8 Hz
11a	135.1, C		137.7, C	
12	189.2, C		188.0, C	
12a	118.7, C		119.4, C	
12b	118.5, C		117.8, C	
1′	71.7, CH	5.73, d, J = 10.0 Hz	72.2, CH	5.67, d, J = 11.6 Hz
2′a	31.7, CH_2	2.31, d, J = 13.7 Hz	34.9, CH_2	2.22, d, J = 12.8 Hz
2′b	31.7, CH_2	2.55, d, J = 12.3 Hz	34.9, CH_2	2.50, q, J = 11.8 Hz
3′	62.2, CH	3.35, t, J = 13.1 Hz	55.6, CH	3.44, d, J = 11.2 Hz
3′-N-CH_3	30.5, CH_3	2.71, s		
4′	72.9, CH	3.75, d, J = 9.3 Hz	74.7, CH	3.56, m
5′	77.9, CH	3.67, p, J = 6.0 Hz	79.3, CH	3.46, d, J = 9.7 Hz
5′-CH_3	18.4, CH_3	1.52, s	18.6, CH_3	1.44, d, J = 6.0 Hz

Summary of NMR data and HRESI-MS data for compounds **3-6**

The HRESI-MS data of compound **3** showed $[M+H]^+$=339.0858，$[M+Na]^+$=361.0690, the molecular formula of **3** was established as $C_{19}H_{14}O_6$, ^1H NMR ($CDCl_3$, 700 MHz,) *δ* 12.28 (s, 1H), 11.64 (s, 1H), 7.68-7.62 (m, 2H), 7.26 (d, *J* = 8.0 Hz, 1H), 6.99 (s, 1H), 3.44 (s, 1H), 3.08 (s, 2H), 3.01 (d, *J* = 15.0 Hz, 1H), 2.97 (d, *J* = 15.0 Hz, 1H), 1.49 (s, 3H); ^{13}C NMR (176 MHz, $CDCl_3$) δ 196.9, 193.0, 183.2, 163.7, 162.0, 150.7, 138.0, 137.8, 135.4, 128.9, 124.0, 122.0, 119.5, 117.5, 115.0, 72.8, 53.8, 45.7, 30.0. The structure of compound **3** was elucidated by comparing the data with previous literature, they were identified as rebelomycin (**3**).

The HRESI-MS data of compound **4** showed $[M+H]^+$=425.2066，$[M+Na]^+$=447.1883, the molecular formula of **4** was established as $C_{24}H_{28}N_2O_5$，^1H NMR (500 MHz, $CDCl_3$) *δ* 8.35 (dd, *J* = 8.7, 1.1 Hz, 1H), 7.87 (dd, *J* = 8.6, 7.0 Hz, 1H), 7.80 (dd, *J* = 8.6, 7.0 Hz, 1H), 7.74 (d, *J* = 6.6 Hz, 1H), 5.45 (t, *J* = 7.8 Hz, 1H), 5.38 (d, *J* = 8.4 Hz, 1H).^{13}C NMR (126 MHz, $CDCl_3$) *δ* 175.3, 166.9, 144.2, 141.5, 140.8, 140.6, 138.4, 133.2, 132.3, 131.5, 130.5, 130.1, 129.7, 129.5, 75.6, 53.5, 52.8, 51.4, 31.6, 29.2, 28.6, 27.6, 22.6, 14.0. The structure of compound **3** was elucidated by comparing the data with previous literature, they were identified as streptophenazine O (**4**).

The HRESI-MS data of compound **5** showed $[M+H]^+$=439.2226，$[M+Na]^+$=461.2038, the molecular formula of **5** was established as $C_{25}H_{30}N_2O_5$, ^1H NMR (500 MHz, $CDCl_3$) *δ δ* 8.39 (dd, *J* = 8.7, 1.5 Hz, 1H), 8.29 (m, 1H), 8.27 (d, *J* = 1.5 Hz, 1H), 7.91 (dd, *J* = 8.7, 6.9 Hz, 1H), 7.83 (dd, *J* = 8.7, 6.8 Hz, 1H), 7.78 (d, *J* = 5.5 Hz, 1H), 5.48 (d, *J* = 7.6 Hz, 1H), 4.95 (s, 1H), 4.14 (s, 3H), 4.01 (s, 1H), 3.89 (s, 3H), 1.58 (s, 2H), 1.57 (s, 2H), 1.55 (s, 1H), 1.28 (s, 4H), 0.85 (d, *J* = 2.6 Hz, 3H), 0.84 (d, *J* = 2.6 Hz, 3H). ^{13}C NMR (126 MHz, $CDCl_3$) δ 175.3, 166.9, 144.2, 141.5, 140.8, 138.4, 133.1, 132.3, 131.5, 130.5, 130.1, 129.7, 129.5, 128.1, 75.6, 53.5, 52.8, 51.4, 38.7, 28.6, 27.9, 27.9, 27.3, 22.6, 22.5. The structure of compound **5** was elucidated by comparing the data with previous literature, they were identified as streptophenazine M (**5**).

The HRESI-MS data of compound **6** showed $[M+H]^+$=383.2424, the molecular formula of **6** was established as $C_{21}H_{24}O_6$, ^1H NMR (500 MHz, $CDCl_3$) *δ* 5.02 (m, 1H), 4.05 (q, *J* = 7.5 Hz, 2H), 3.87 (d, *J* = 7.2 Hz, 2H), 2.54 (d, *J* = 3.8 Hz, 1H), 2.07-1.89 (m, 1H), 1.85-1.72 (m, 3H), 1.64 (q, *J* = 5.0 Hz, 1H), 1.61-1.46 (m, 1H), 1.25 (d, *J* = 6.2 Hz, 3H), 1.11 (t, *J* = 7.3 Hz, 7H), 0.90 (t, *J* = 7.4 Hz, 3H). ^{13}C NMR (126 MHz, $CDCl_3$) *δ* 174.5, 174.2, 80.0, 79.9, 76.4, 76.3, 73.3, 69.1, 45.3, 45.1, 42.3, 39.9, 31.5, 31.4, 28.2, 28.0, 27.4, 20.5, 13.2, 12.6, 9.3. The structure of compound **6** was elucidated by comparing the data with previous literature, they were identified as dimeric dynactin (**6**).

Table S6. High resolution MS (HRMS), retention time, and predicted chemical formula for nodes in streptophenazine cluster from GNPS molecular network. (Related to **Figure 3** and **Figure 5**)

**Precursor ion mass from molecular network**	**Determined m/z**	**Retention time (min)**	**Predicted formula**	**Predicted Structure**
383.2	383.161	18.6	$C_{21}H_{23}N_2O_5^+$	lacked methylene unit from 397.2
397.2	397.176	20.0	$C_{22}H_{25}N_2O_5^+$	streptophenazine E (**42**)
411.2	411.191	21.7	$C_{23}H_{27}N_2O_5^+$	streptophenazine C (**41**)
				streptophenazine D (**31**)
				streptophenazine P (**40**)
423.2	423.191	24.1	$C_{24}H_{27}N_2O_5^+$	oxo-streptophenazine A (**45**)
				oxo-streptophenazine B (**46**)
425.2	425.172	13.9	$C_{24}H_{29}N_2O_5^+$	streptophenazine A (**29**)
				streptophenazine B (**30**)
	425.210	6.6	$C_{24}H_{29}N_2O_5^+$	streptophenazine O (**4**)
437.2	437.209	26.3	$C_{25}H_{29}N_2O_5^+$	oxo-streptophenazine G (**24**)
				oxo-streptophenazine F (**25**)
439.2	439.226	8.6	$C_{25}H_{31}N_2O_5^+$	streptophenazine M (**5**)
	439.187	15.9	$C_{25}H_{31}N_2O_5^+$	diastereomer at C-2' of streptophenazine G (**49**)
				diastereomer at C-2' of streptophenazine F (**50**)
	439.231	0.7	$C_{25}H_{31}N_2O_5^+$	streptophenazine G (**34**)
				streptophenazine F (**35**)
443.2	443.164	19.5		
453.2	453.203	11.6	$C_{29}H_{33}N_2O_5^+$	streptophenazine L (**39**)
	453.237	26.4	$C_{29}H_{33}N_2O_5^+$	diastereomer of streptophenazine L
467.2	467.181	14.4	$C_{26}H_{31}N_2O_6^+$	streptophenazine K (**38**)
	467.217	21.9	$C_{26}H_{31}N_2O_6^+$	streptophenazine J (**37**)
467.3	467.254	28.0	$C_{26}H_{31}N_2O_6^+$	diastereomer of streptophenazine K or J
481.2	481.197	17.8	$C_{27}H_{33}N_2O_6^+$	additional methylene unit from 467.2

Figure S1. The whole genome phylogenetic tree of marine-derived SCSIO LCY30

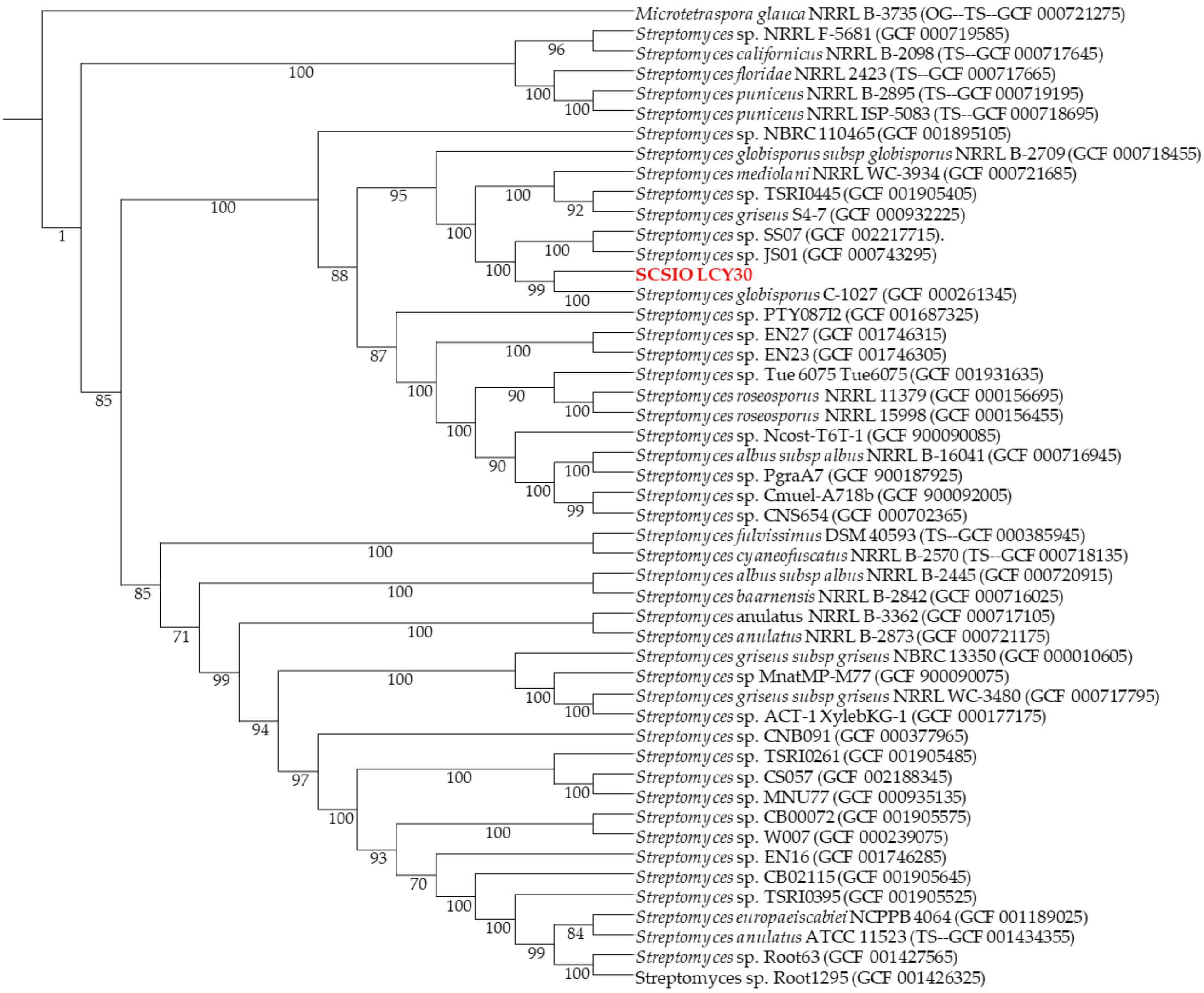

Figure S2. Molecular network showing production of streptophenazines, related to **Figure 7**.

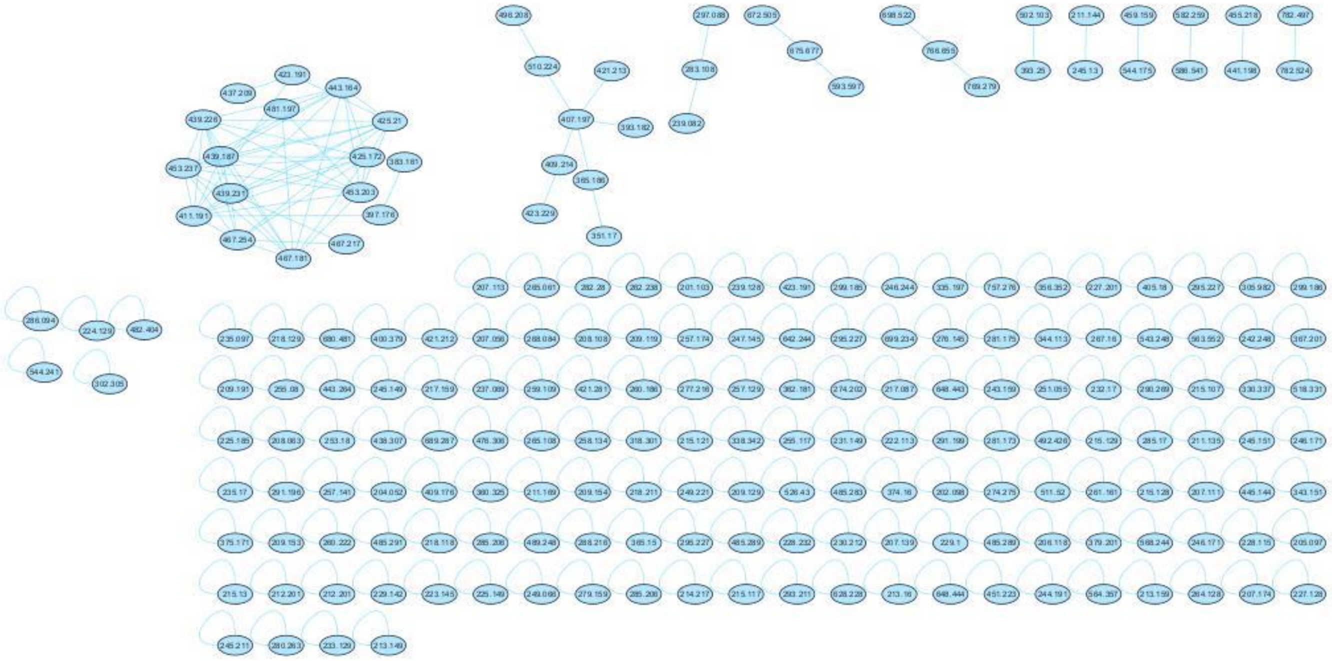

Figure S3. Structures of streptophenazines.

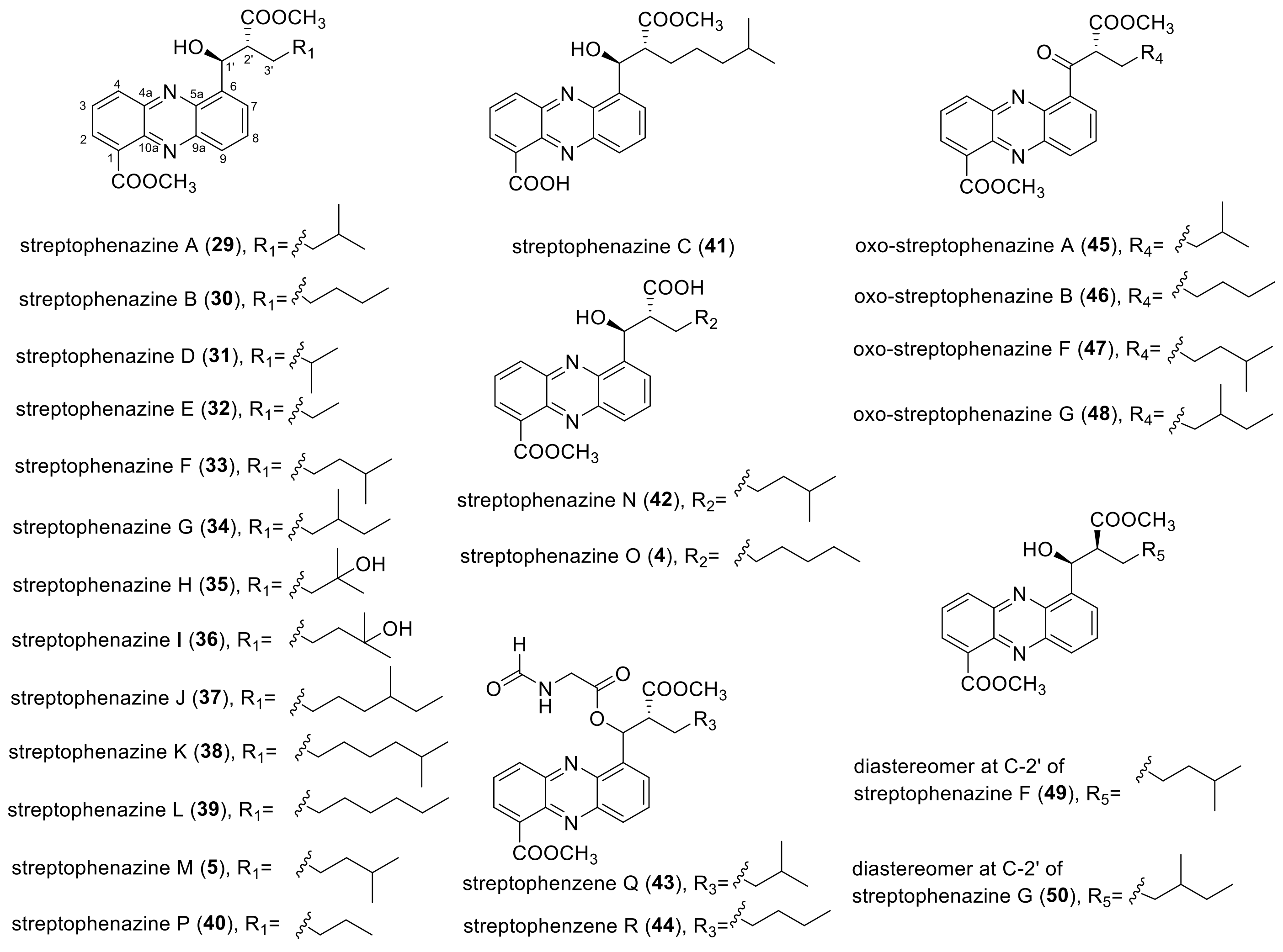

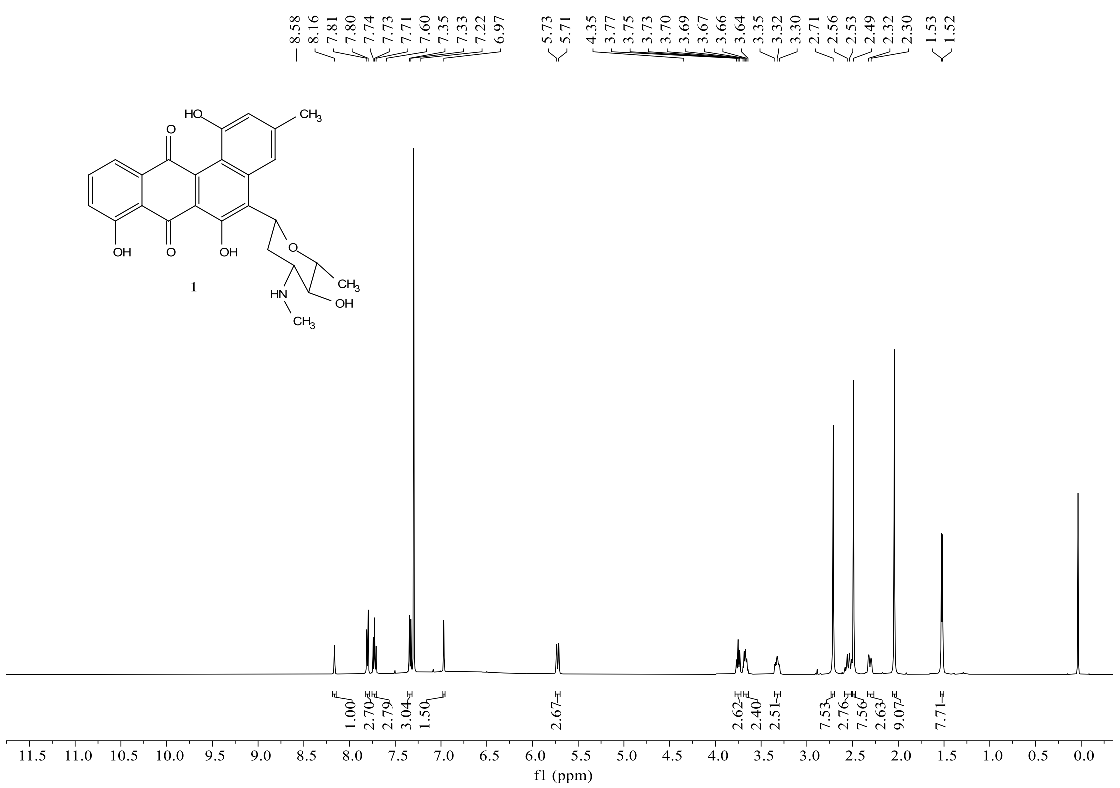

Figure S4. ^{1}H NMR (500 MHz, Chloroform-*d*) spectrum of **1**

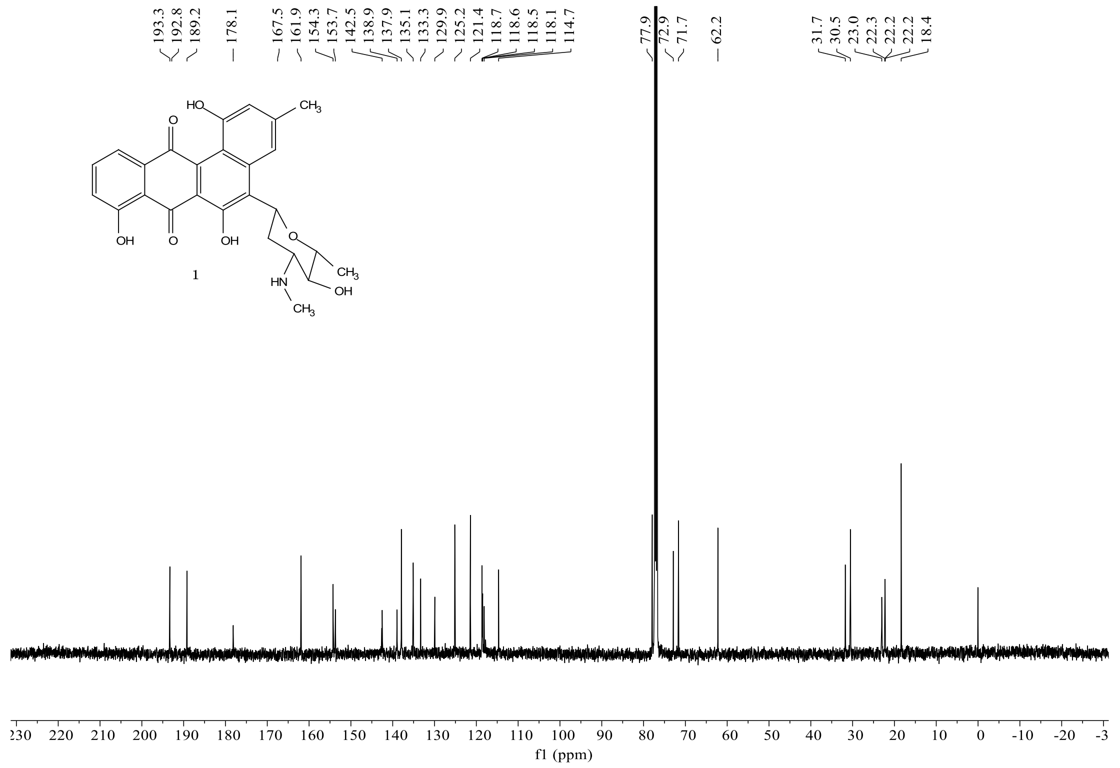

Figure S5. ^{13}C NMR (176MHz, Chloroform-*d*) spectrum of **1**

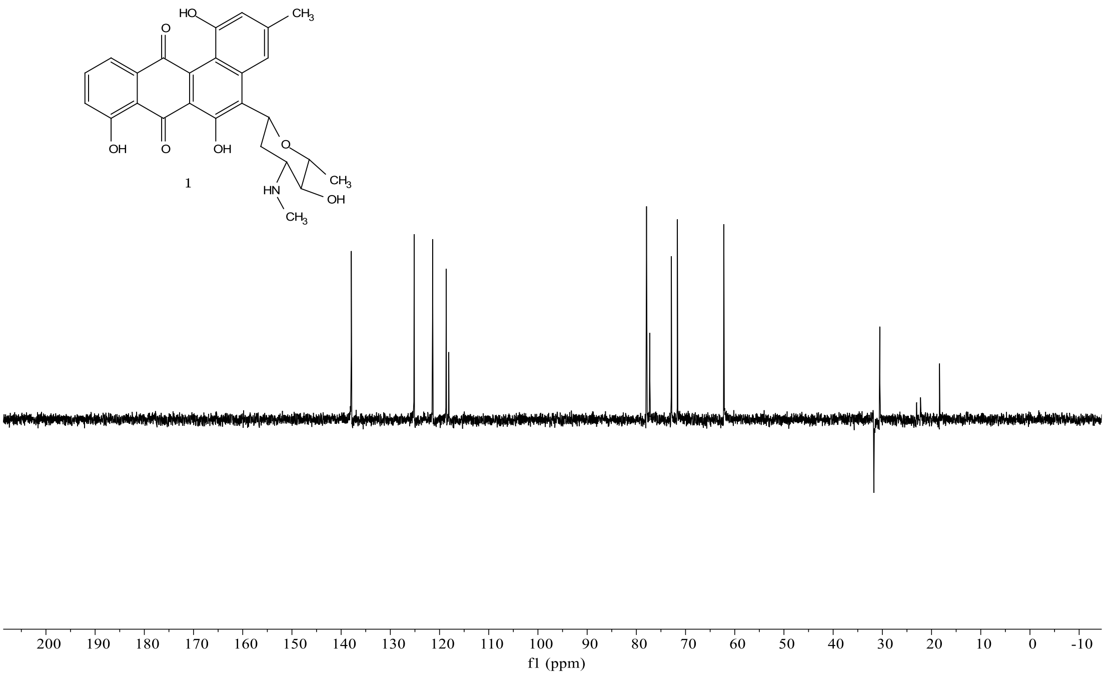

Figure S6. DEPT135 spectrum of **1**

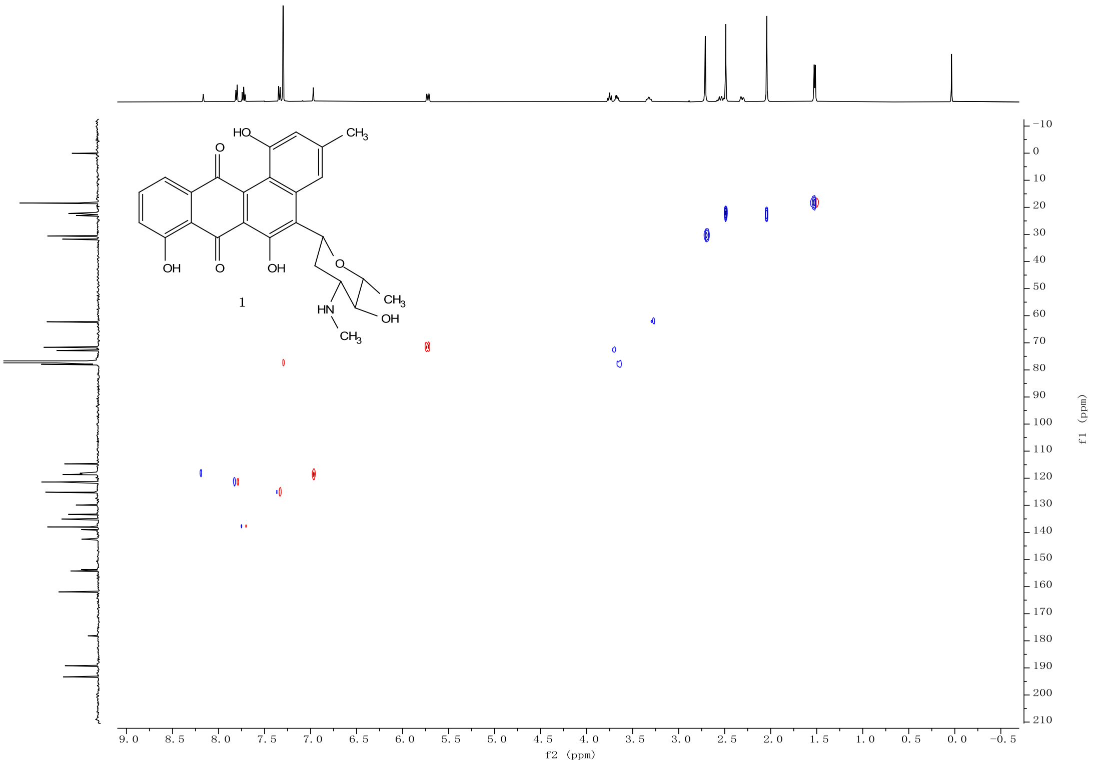

Figure S7. HSQC spectrum of **1**

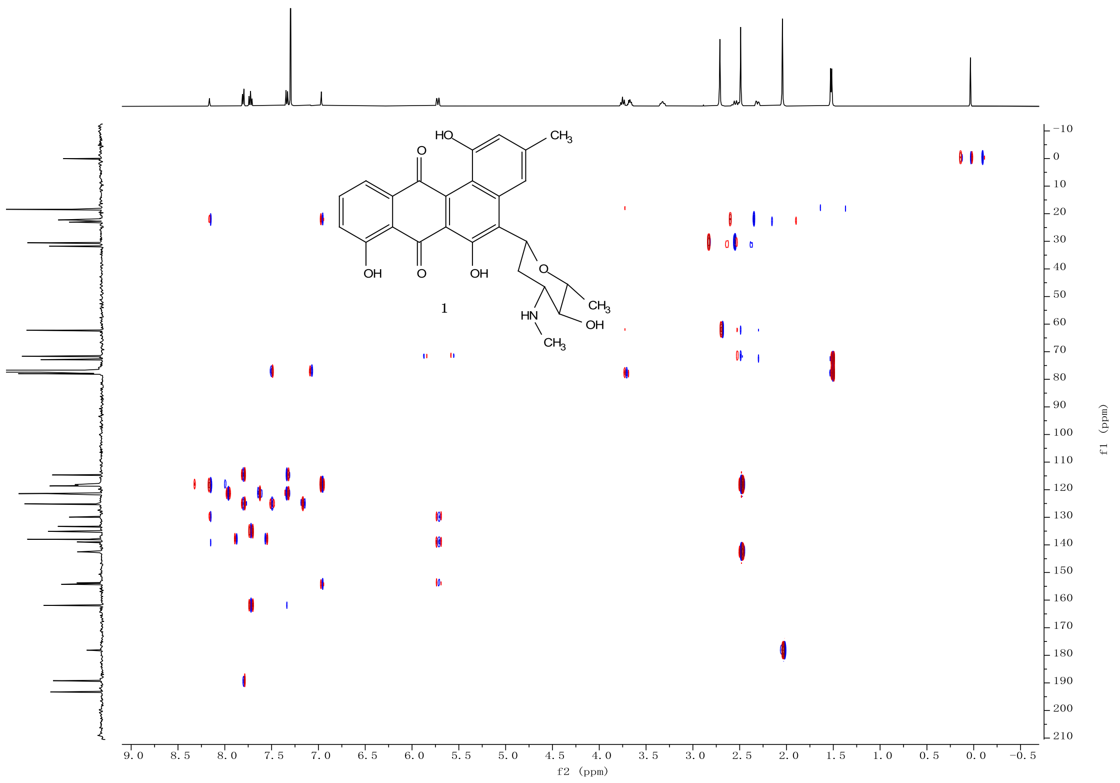

Figure S8. HMBC spectrum of **1**

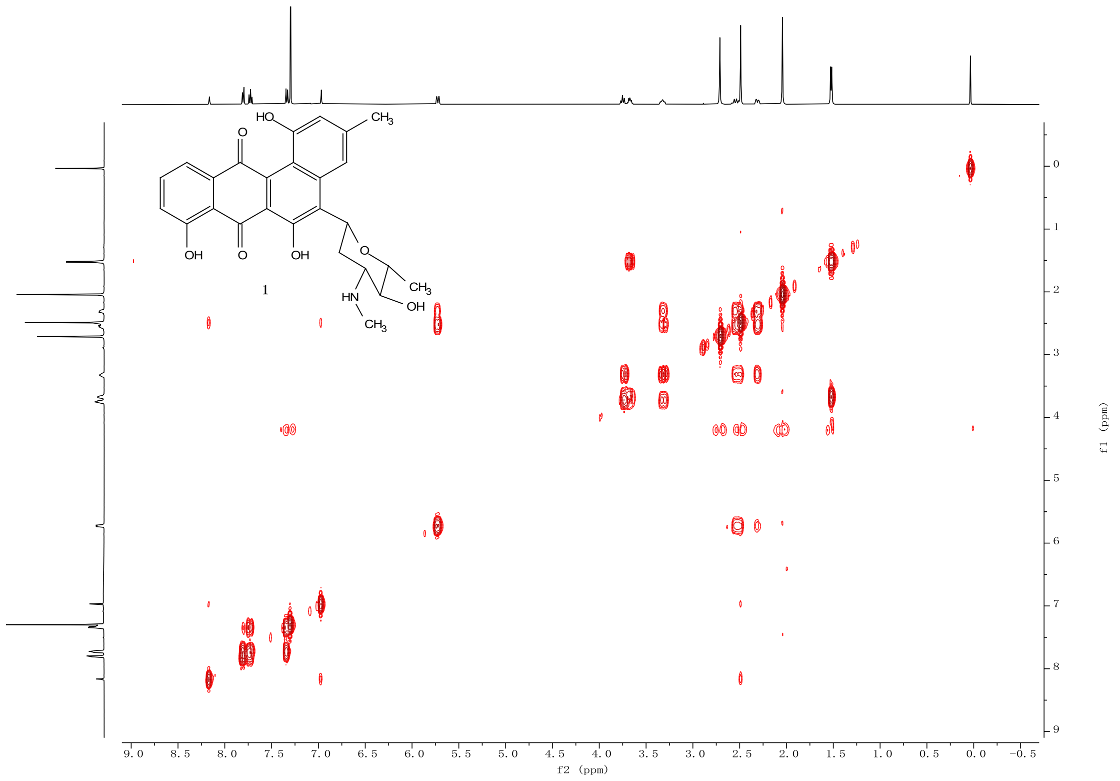

Figure S9. ^{1}H-^{1}H COSY spectrum of **1**

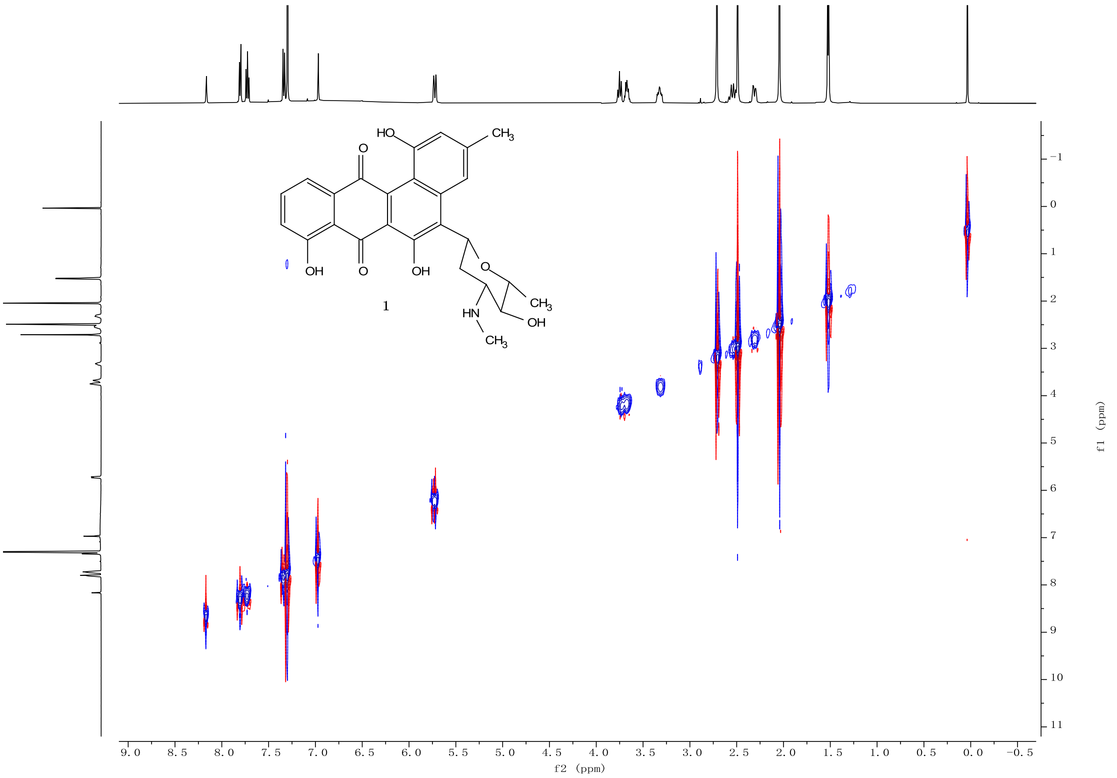

Figure S10. NOESY spectrum of **1**

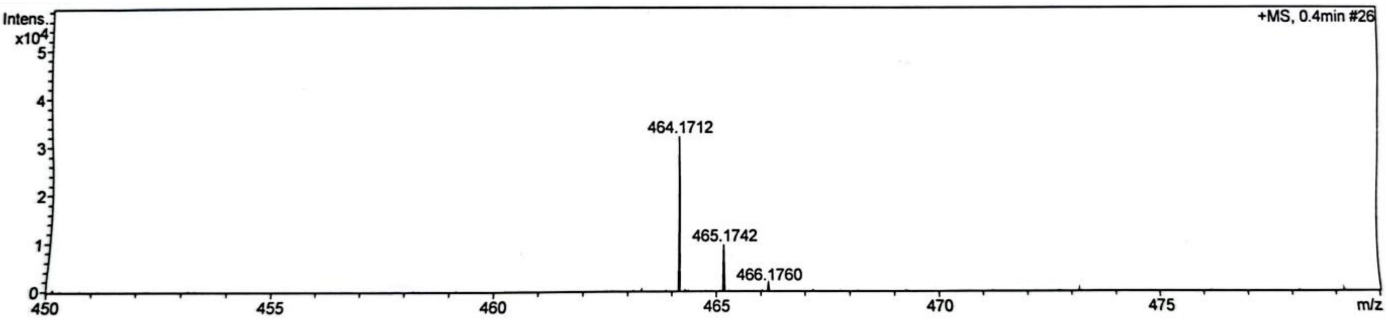

Meas. m/z	#	Ion Formula	Score	m/z	err [ppm]	err [mDa]	mSigma	rdb	e⁻ Conf	N-Rule
464.1712	1	C26H26NO7	100.00	464.1704	-1.8	-0.8	6.7	14.5	even	ok
927.3353	1	C52H51N2O14	100.00	927.3335	-1.9	-1.8	15.4	28.5	even	ok

Figure S11. HRESI-MS spectrum of **1**

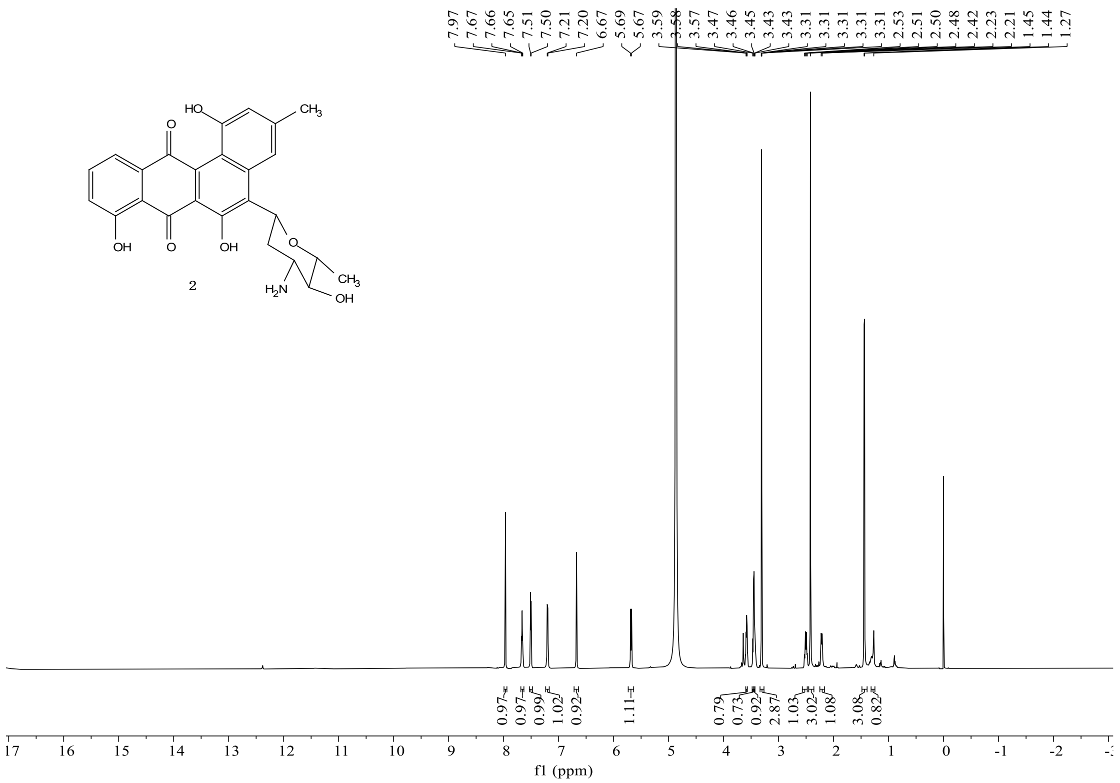

Figure S12. ^{1}H NMR (500 MHz, MeOD) spectrum of **2**

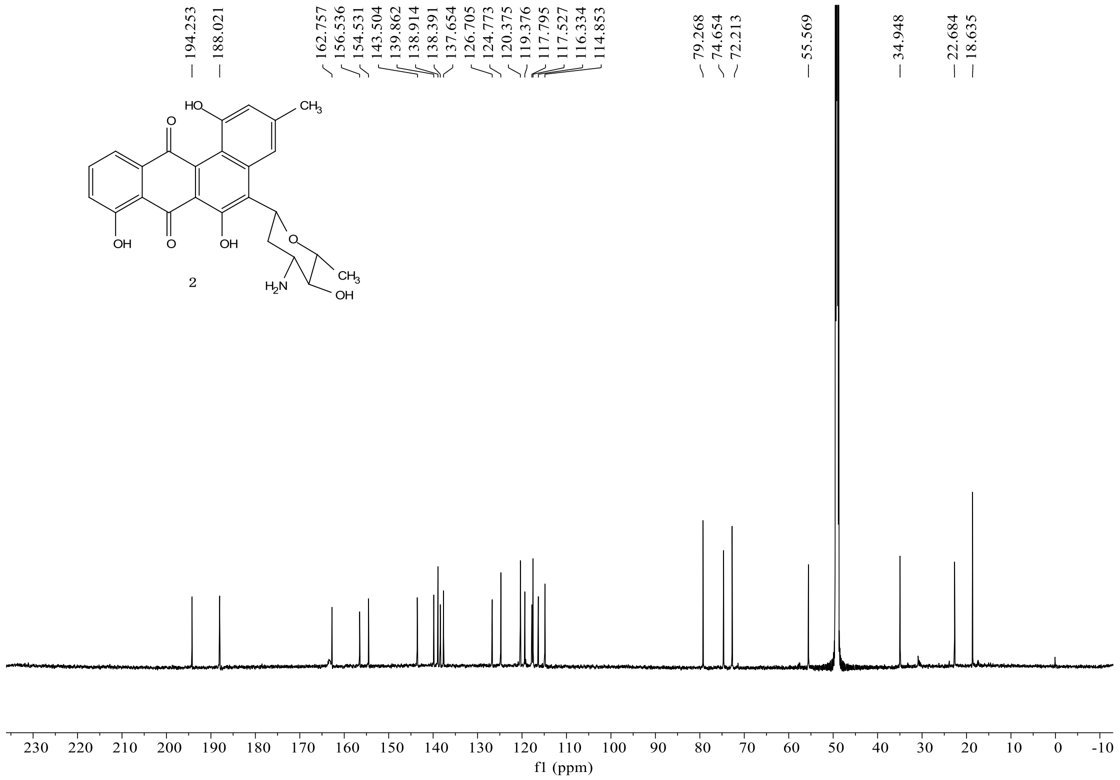

Figure S13. ^{13}C NMR (176MHz, MeOD) spectrum of **2**

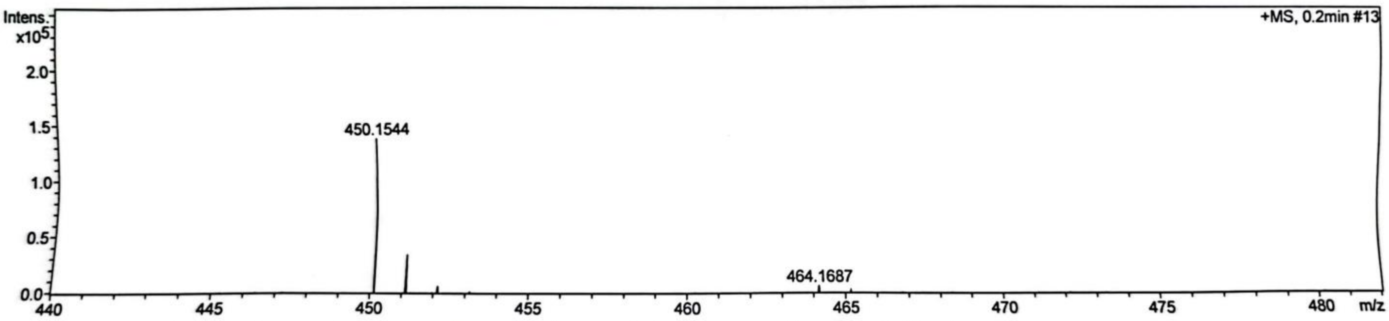

Meas. m/z	#	Ion Formula	Score	m/z	err [ppm]	err [mDa]	mSigma	rdb	e⁻ Conf	N-Rule
450.1544	1	C25H24NO7	100.00	450.1547	0.7	0.3	14.7	14.5	even	ok
472.1347	1	C25H23NNaO7	100.00	472.1367	4.2	2.0	21.5	14.5	even	ok
899.3019	1	C50H47N2O14	100.00	899.3022	-0.3	-0.3	17.4	28.5	even	ok
921.2818	1	C50H46N2NaO14	100.00	921.2841	-2.5	-2.3	40.6	28.5	even	ok

Figure S14. HRESI-MS spectrum of **2**

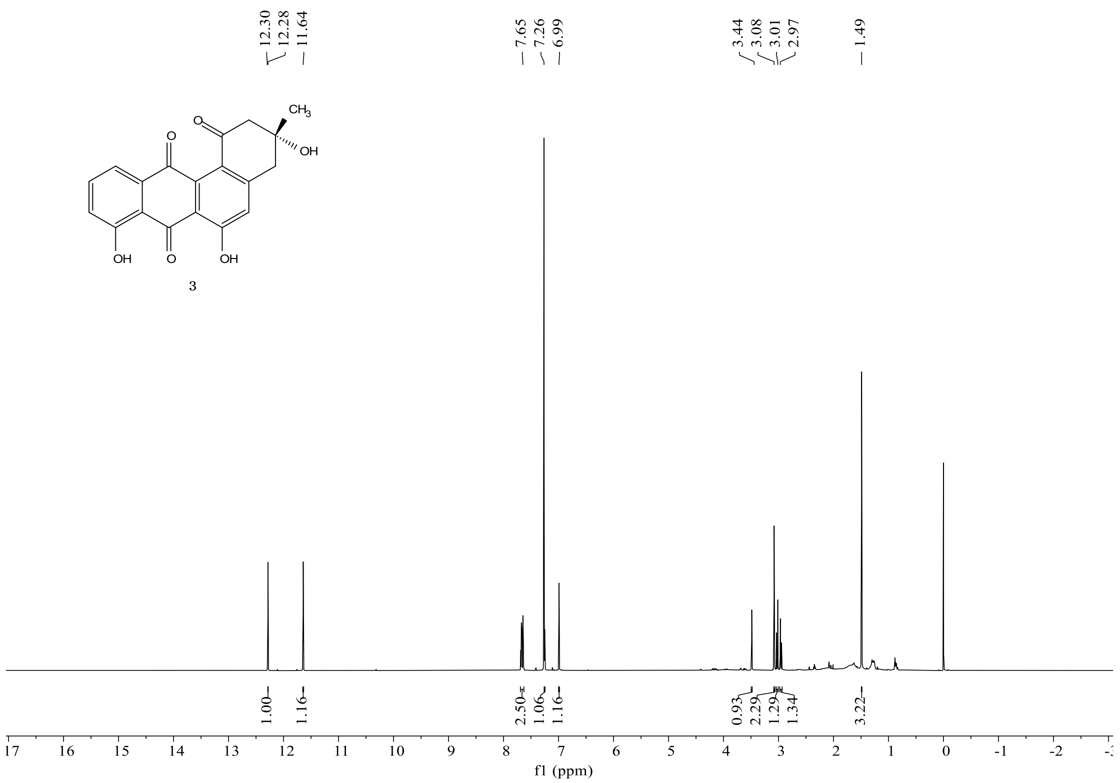

Figure S15. ^{1}H NMR (700 MHz, Chloroform-*d*) spectrum of **3**

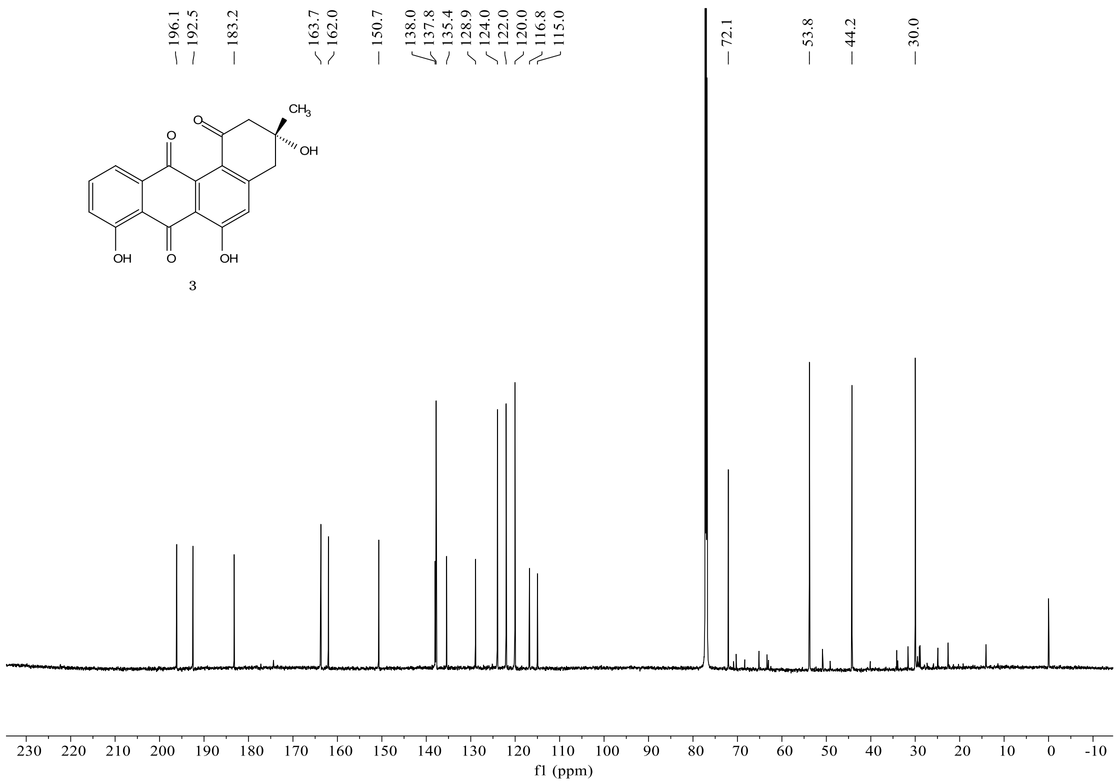

Figure S16. ^{13}C NMR (176MHz, Chloroform-*d*) spectrum of **3**

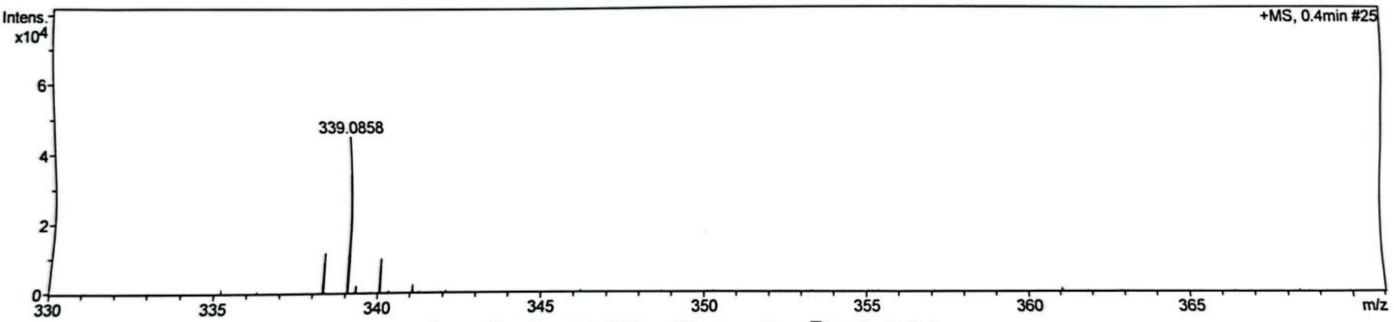

Meas. m/z	#	Ion Formula	Score	m/z	err [ppm]	err [mDa]	mSigma	rdb	e⁻ Conf	N-Rule
339.0858	1	C19H15O6	100.00	339.0863	-1.4	-0.5	5.2	12.5	even	ok
361.0690	1	C19H14NaO6	99.47	361.0683	1.9	0.7	18.0	12.5	even	ok
699.1461	1	C38H28NaO12	100.00	699.1473	-1.7	-1.2	51.1	24.5	even	ok

Figure S17. HRESI-MS spectrum of **3**

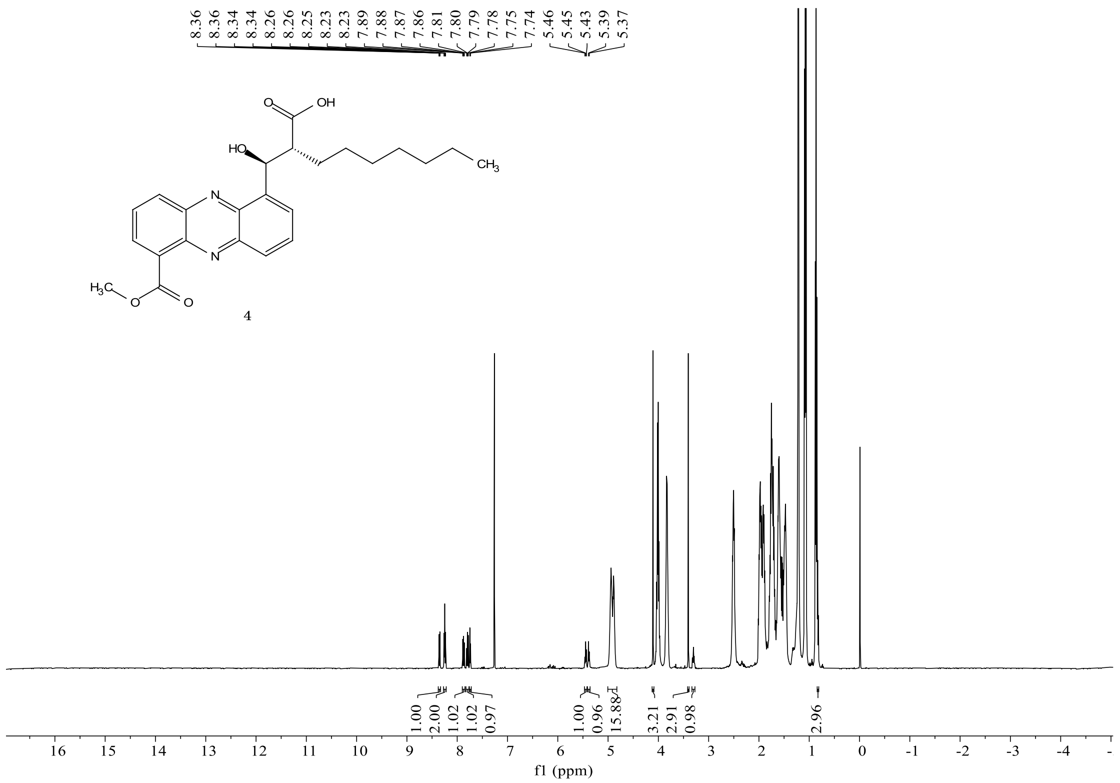

Figure S18. ^{1}H NMR (700 MHz, Chloroform-*d*) spectrum of **4**

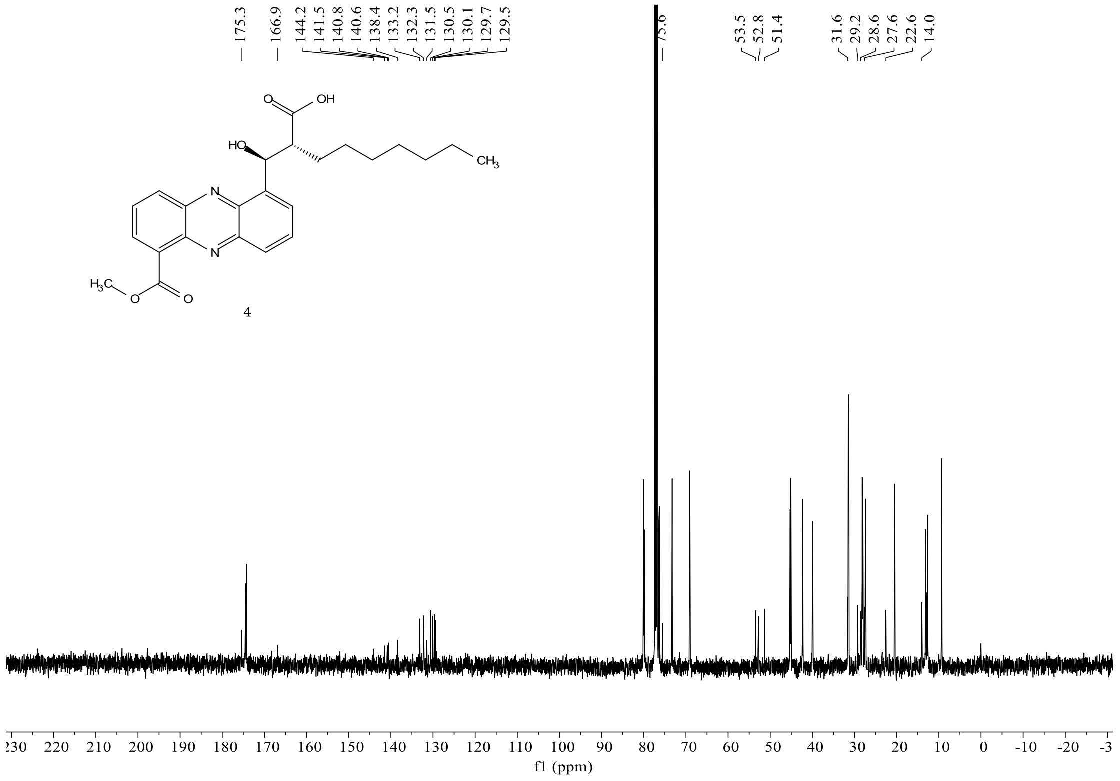

Figure S19. ^{13}C NMR (176MHz, Chloroform-*d*) spectrum of **4**

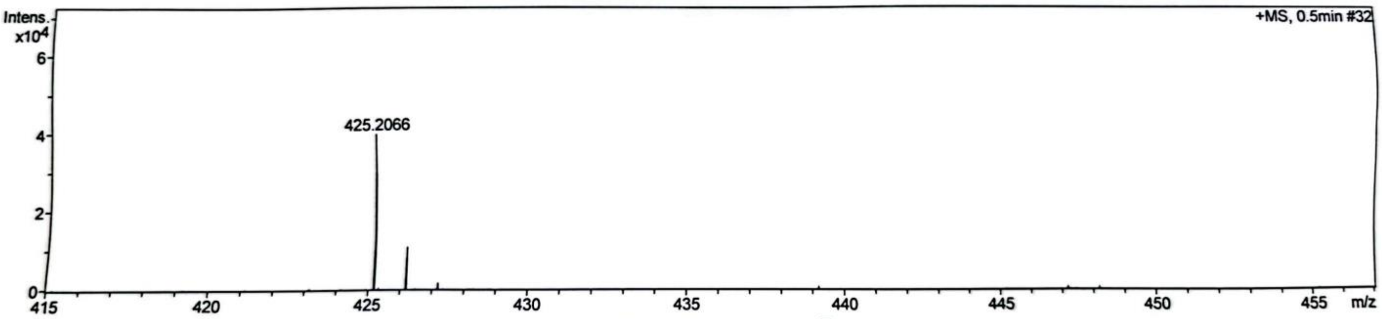

Meas. m/z	#	Ion Formula	Score	m/z	err [ppm]	err [mDa]	mSigma	rdb	e⁻ Conf	N-Rule
425.2066	1	C24H29N2O5	100.00	425.2071	-1.2	-0.5	2.0	11.5	even	ok
447.1883	1	C24H28N2NaO5	100.00	447.1890	1.6	0.7	151.9	11.5	even	ok

Figure S20. HRESI-MS spectrum of **4**

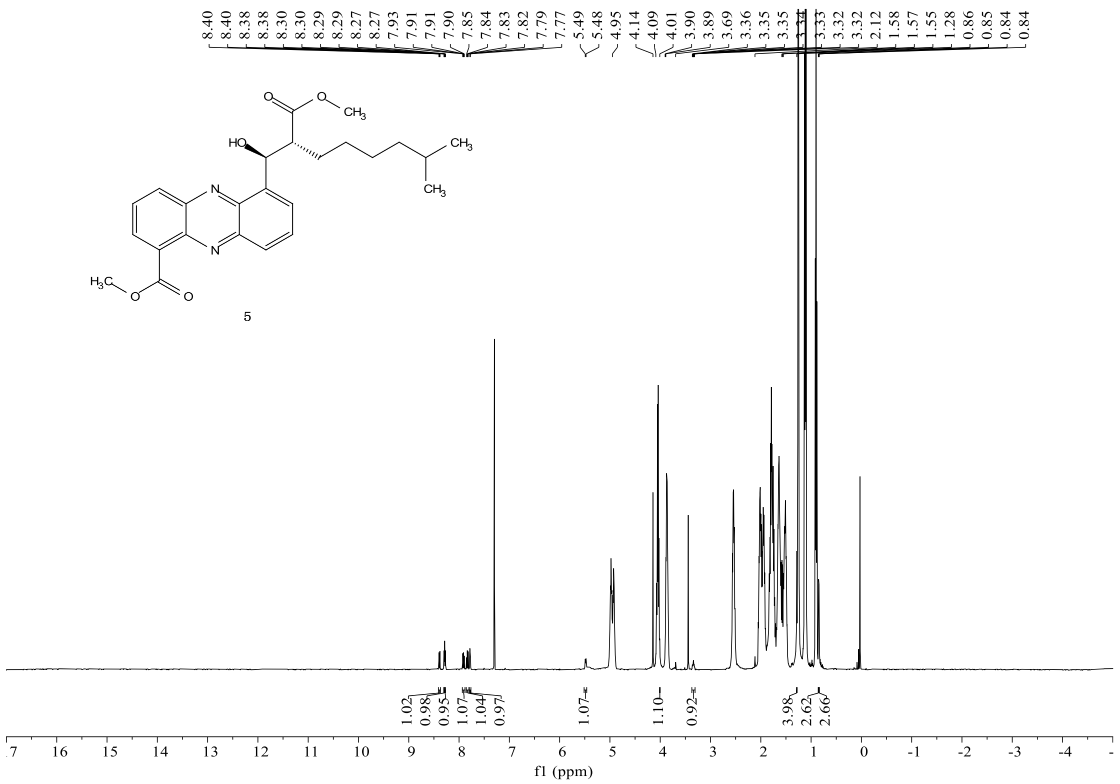

Figure S21. ^{1}H NMR (700 MHz, Chloroform-*d*) spectrum of **5**

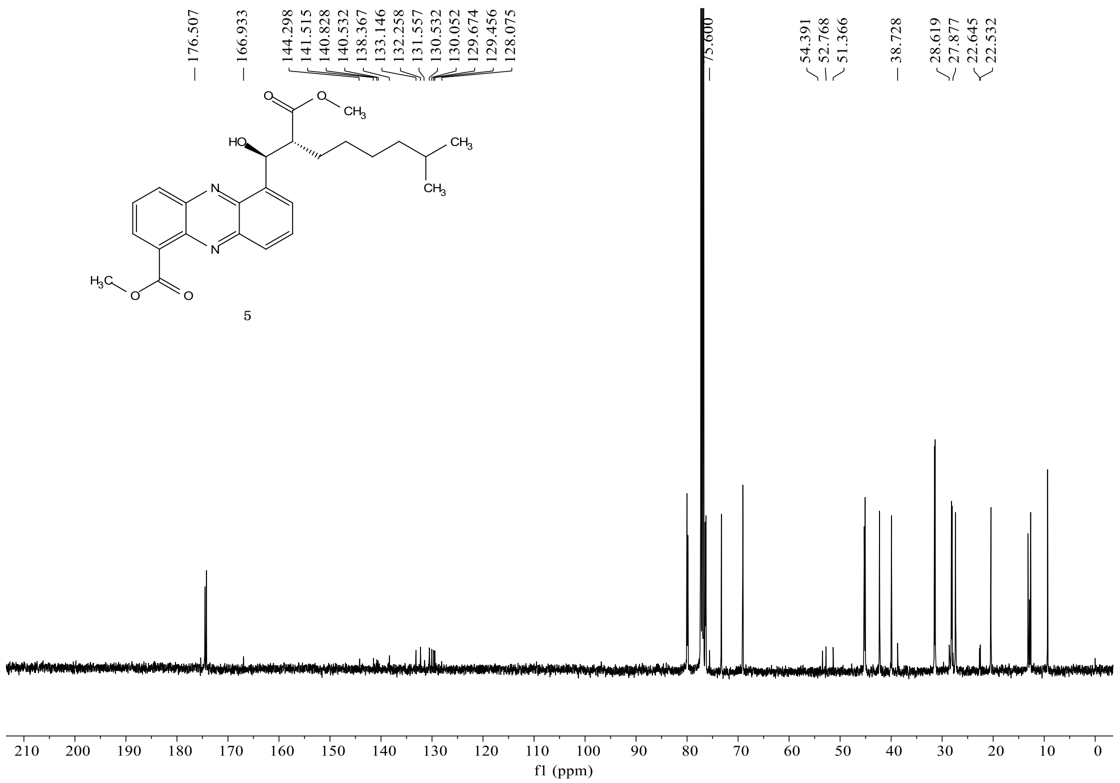

Figure S22. ^{13}C NMR (176MHz, Chloroform-*d*) spectrum of **5**

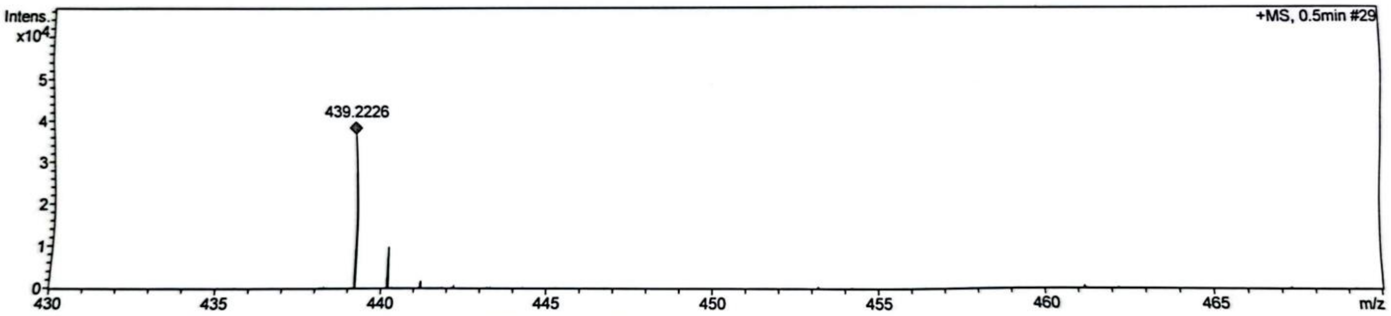

Meas. m/z	#	Ion Formula	Score	m/z	err [ppm]	err [mDa]	mSigma	rdb	e⁻ Conf	N-Rule
439.2226	1	C25H31N2O5	100.00	439.2227	0.3	0.1	8.7	11.5	even	ok
461.2038	1	C25H30N2NaO5	100.00	461.2047	-2.0	-0.9	96.6	11.5	even	ok

Figure S23. HRESI-MS spectrum of **5**

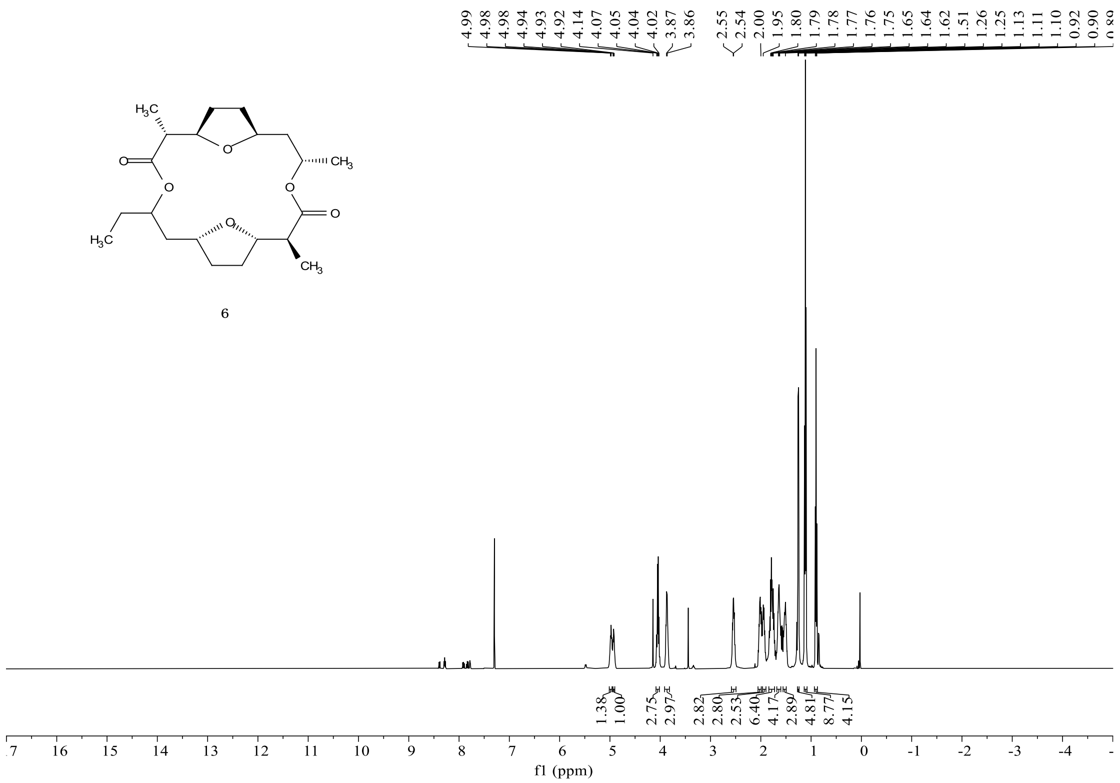

Figure S24. ^{1}H NMR (700 MHz, Chloroform-*d*) spectrum of **6**

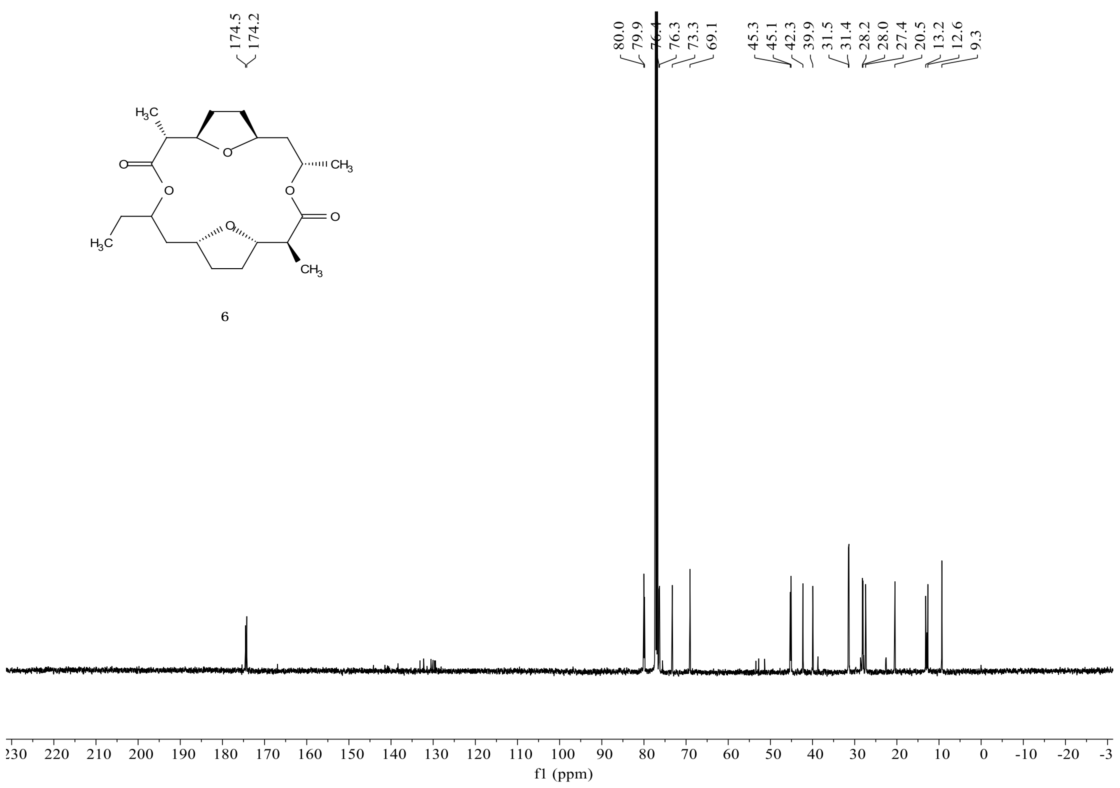

Figure S25. ^{13}C NMR (176MHz, Chloroform-*d*) spectrum of **6**
